# Esophageal squamous cell carcinoma transcriptome reveals the effect of *FOXM1* on patient outcome through novel PIK3R3 mediated activation of PI3K signaling pathway

**DOI:** 10.18632/oncotarget.24621

**Published:** 2018-03-30

**Authors:** Pedro Nicolau-Neto, Nathalia Meireles Da Costa, Paulo Thiago de Souza Santos, Isabela Martins Gonzaga, Maria Aparecida Ferreira, Simone Guaraldi, Miguel Angelo Moreira, Hector N. Seuánez, Lilian Brewer, Anke Bergmann, Mariana Boroni, Andre Luiz Mencalha, Cleber Dario Pinto Kruel, Sheila Coelho Soares Lima, Dominic Esposito, Tatiana Almeida Simão, Luis Felipe Ribeiro Pinto

**Affiliations:** ^1^ Molecular Carcinogenesis Program, Instituto Nacional de Câncer (INCA), Rio de Janeiro, 20231-050 RJ, Brasil; ^2^ Endoscopy Section, Instituto Nacional de Câncer (INCA), Praça Cruz Vermelha, 20230-130 RJ, Brasil; ^3^ Genetic Program, Instituto Nacional de Câncer (INCA), Rio de Janeiro, 20231-050 RJ, Brasil; ^4^ Biochemistry Department, Instituto de Biologia Roberto Alcântara Gomes, Universidade do Estado do Rio de Janeiro, Rio de Janeiro, 20551-030 RJ, Brasil; ^5^ Biophysics and Biometry Department, Instituto de Biologia Roberto Alcântara Gomes, Universidade do Estado do Rio de Janeiro, Rio de Janeiro, 20551-030 RJ, Brasil; ^6^ Surgery Department, Faculty of Medical Sciences, Universidade Federal do Rio Grande do Sul, Porto Alegre, 90035-003 RS, Brasil; ^7^ Cancer Research Technology Program, Frederick National Laboratory for Cancer Research, Leidos Biomedical Research, Inc., Frederick, 21701 MD, USA

**Keywords:** esophageal squamous cell carcinoma, FOXM1, PIK3R3, PI3K/AKT pathway, target therapy

## Abstract

Esophageal squamous cell carcinoma (ESCC) presents poor prognosis, and patients diagnosed with this tumor currently lack target treatments. Therefore, in order to identify potential targets for ESCC treatment, we carried out a transcriptome analysis with ESCC and paired nonmalignant surrounding mucosa samples, followed by a master regulator analysis, and further explored the role of the identified central regulatory genes through *in vivo* and *in vitro* assays. Among the transcription factors deregulated/enriched in ESCC, we focused on FOXM1 because of its involvement in the regulation of critical biological processes. A new transcriptome analysis performed with ESCC cell lineage TE-1 showed that the modulation of *FOXM1* expression resulted in *PIK3R3* expression changes, whereas chromatin immunoprecipitation assay revealed that FOXM1 was capable of binding onto *PIK3R3* promoter, thus demonstrating that *PIK3R3* is a new FOXM1 target. Furthermore, FOXM1 overexpression resulted in the activation of PIK3/AKT signaling pathway through PIK3R3-mediated AKT phosphorylation. Finally, the analysis of the clinic-pathological data of ESCC patients revealed that overexpression of both FOXM1 and PIK3R3 was associated with poor prognosis, but only the latter was an independent prognosis factor for ESCC patients. In conclusion, our results show that FOXM1 seems to play a central role in ESCC carcinogenesis by upregulating many oncogenes found overexpressed in this tumor. Furthermore, *PIK3R3* is a novel FOXM1 target that triggers the activation of the PI3K/AKT pathway in ESCC cells.

## INTRODUCTION

Esophageal cancer (EC) is the sixth most frequent cause of cancer-related death worldwide [1]. Squamous cell carcinoma (ESCC) is the main EC histotype, accounting for approximately 80% of all cases worldwide [2]. As ESCC is often diagnosed at a late stage, the majority of patients fail to benefit from the commonly employed therapeutic procedures, resulting in a 5-year survival rate below 15% [3–5]. Currently there is no approved target therapy for ESCC treatment, since there is limited knowledge about the main molecular alterations present in this tumor [6].

Recent technologies such as microarray analysis provide powerful tools for understanding tumor biology due to their capacity of simultaneously analyzing thousands of genes. DNA microarray analysis allowed a molecular classification and the identification of target drivers of breast, [7], lung [8], and colorectal [9] cancer.

This study was designed to analyze ESCC global gene expression with the aim of contributing the understanding of this tumor biology, revealing significant altered signaling pathways and, consequently, potential druggable targets. Among the main transcription factors responsible for the differentially expressed genes in ESCC, Forkhead box M1 (*FOXM1*) was shown to have a central role in cell signaling pathways, while Phosphoinositide-3-Kinase Regulatory Subunit 3 (*PIK3R3*) was characterized as a novel FOXM1 target. Furthermore, modulation of FOXM1 expression in the ESCC cell line, TE-1, resulted in activation of the PI3K/AKT pathway through PIK3R3 overexpression. Finally, *PIK3R3* expression was shown to be an independent prognostic biomarker for this tumor.

## RESULTS

### Determination of ESCC global gene expression profile

**Figure 1:**
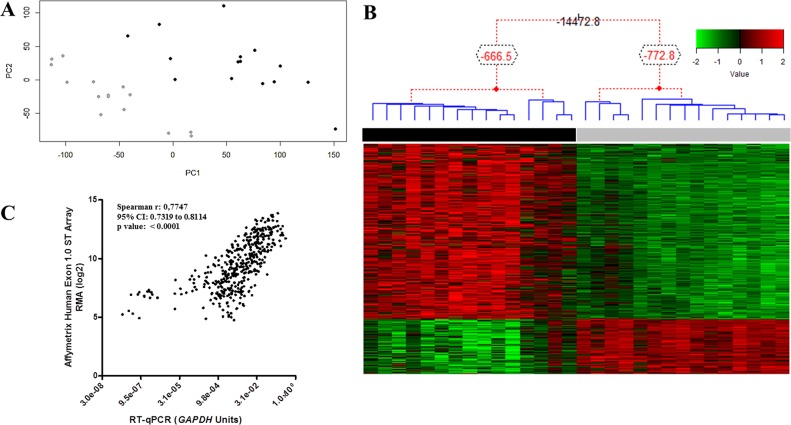
ESCC global gene expression profile **(A)** Two sample groups were observed in this unsupervised analysis, which considered all probe sets present on the microarray chip. The groups respected the histologic classification of samples. PC1, principal component 1; PC2, principal component 2. **(B)** Normalized expression data of the 1,328 DEGs were used for clustering analysis. Hierarchical clustering clearly separated tumor (black bar) and nonmalignant surrounding mucosa (gray bar) samples, without misclassification. Each column represents an individual sample, and each line represents a DEG. The red and green colors represent increased and decreased gene expression, respectively. **(C)** Correlation between microarray and RT-qPCR expression data for 15 genes in ESCC tissue compared with nonmalignant surrounding mucosa. Abbreviations: r, Spearman correlation coefficient; CI, confidence interval.

Microarray analysis of 15 paired tumor and NSM samples revealed 1,328 DEG, 994 of which with overexpression in tumor tissue and 334 underexpressed (Supplementary Table 1). Principal component analysis separated tumor from NSM samples (Figure 1A). Unsupervised hierarchical Bayesian clustering, using DEG expression values, also showed two different groups, one comprising ESCC and another of NSM samples (Figure 1B).

Validation of microarray data was carried out by RT-qPCR for thirteen DEG, 11 overexpressed genes (*FOXM1, PLK1, CDK1, AKT3, PIK3R3, CCNB1, HOXD10, ETV5, MMP9, MMP12* and *FSCN1*), two underexpressed genes (*NDRG2* and *CRISP3*), and one gene (*STAT3*) with unchanged expression. All genes showed similar expression profiles to those reported by microarray analysis (r = 0.77; p < 0.001; Figure 1C and Supplementary Figure 1).

### Enrichment of differentially expressed genes

**Figure 2:**
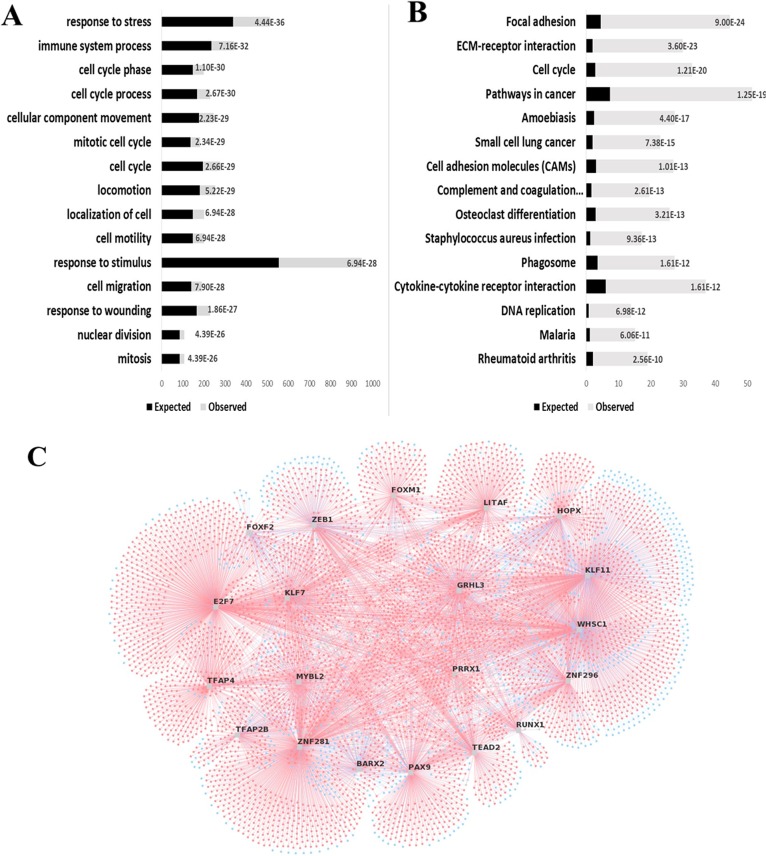
Gene enrichment analyses indicate FOXM1 as an important transcriptional factor in ESCC GOBiological Processes **(A)** or KEGG Pathways **(B)** associated with ESCC overexpressed genes are plotted in graphic bars. Black bars, expected number of DEG in each process or pathway; gray bars, observed number of GDE in each process or pathway; numbers on bars, p values. **(C)** Thetop 20 most relevant master regulators responsible for the ESCC gene expression profile are identified in the figure. Master regulators are plotted randomly and connected to their DEG targets. Blue circles, underexpressed genes; red circles, overexpressed genes.

Enrichment of ESCC overexpressed genes pointed to alterations of biological processes like cell cycle control, response to stress and stimuli, cell motility and division, interaction between cells and extracellular matrix, and immune response (Figure 2A–2B). In addition, enrichment of underexpressed genes involved biological processes related to metabolic pathways.

Prediction of major regulators of gene expression in ESCC identified 176 potential transcription factors regulating the 1,328 DEG herein identified (Figure 2C; Supplementary Table 2), of which 56 were found to be differentially expressed. Among the enriched transcription factors we focused on FOXM1 for further analyses in view of its association with the above mentioned biological processes and cell signaling pathways, and the fact that the MRA algorithm listed 119 genes as FOXM1 targets, six of which expected to be differentially expressed. However, we actually found 70 DEG to be FOXM1-related, accounting for approximately 60% of the listed targets (p = 0.041).

### Analysis of the relevance of *FOXM1* expression in ESCC

**Figure 3:**
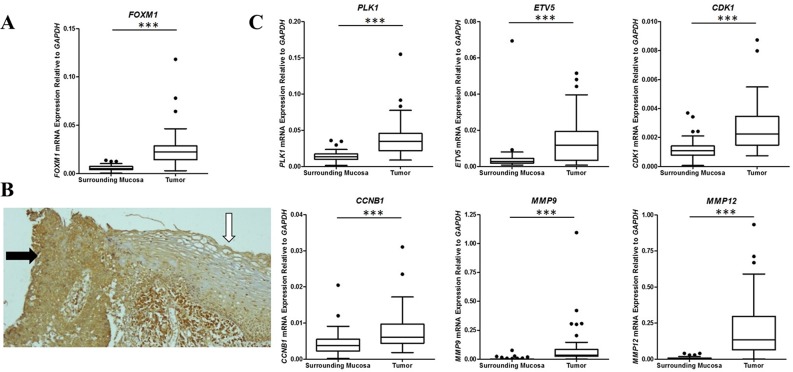
Analysis of expression of *FOXM1* and members of its cell signaling pathway in ESCC and nonmalignant surrounding mucosa **(A)** Box plot showing *FOXM1* expression by RT-qPCR in ESCC and nonmalignant surrounding mucosa. **(B)** FOXM1 protein expression by immunohistochemistry in ESCC (black arrow) and nonmalignant surrounding mucosa (white arrow). **(C)** Box plot showing expression by RT-qPCR of different genes described as regulated by FOXM1 (*PLK1, ETV5*, *CDK1, CCNB1, MMP9,* and *MMP12*) in ESCC and nonmalignant surrounding mucosa. ^***^ p < 0.0001.

*FOXM1* expression was found to be 3.4-fold higher in ESCC than in NSM by microarray analysis. Furthermore, *FOXM1* expression assessed by RT-qPCR in both the investigation set and the validation sample set confirmed microarray data, showing a 4-fold higher expression in tumors (Figure 3A). These results were then confirmed by immunohistochemical analyses that showed a higher FOXM1 expression in neoplastic tissues with respect to NSM (Figure 3B).

Interestingly, our microarray profile expression analysis also revealed that six genes, well known to be regulated by FOXM1, were also overexpressed in ESCC: *PLK1* (2.9-fold), *CDK1* (3.3-fold), *CCNB1* (2.4-fold), *ETV5* (3.3-fold) *MMP9* (5.2-fold), and *MMP12* (28.2-fold). Accordingly, their overexpression was confirmed by RT-qPCR (Figure 3C) and positively correlated with *FOXM1* expression (Supplementary Table 3).

Taken together, these findings strongly pointed to the relevance of *FOXM1*-regulated pathways in ESCC.

### Identification of novel FOXM1 targets in ESCC

**Figure 4:**
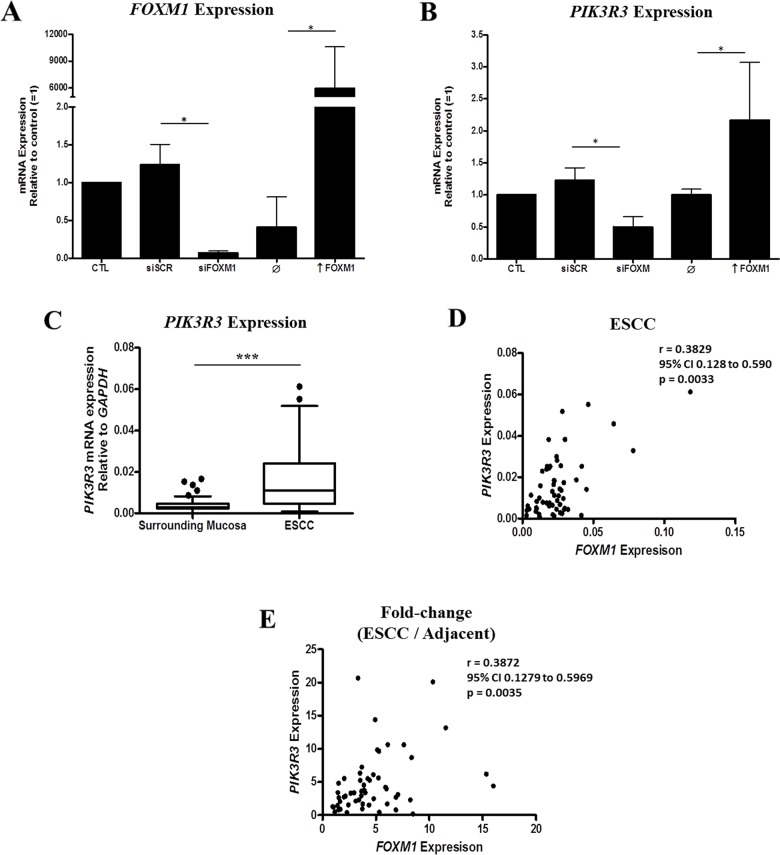
Identification of a positive association between *FOXM1* and *PIK3R3* expression in ESCC **(A)** *FOXM1* expression in TE-1 cell line transfected with siFOXM1 or pcDNA3a-FOXM1 (↑ FOXM1) (n = 3). **(B)** *PIK3R3* expression in TE-1 cell line transfected with siFOXM1 or pcDNA3a-FOXM1 (↑ FOXM1). SCR, scramble; siFOXM1, siRNA against *FOXM1* mRNA; ↑ FOXM1, pcDNA3a vector with FOXM1 gene (n = 3). **(C)** Box plot showing *PIK3R3* expression by RT-qPCR in ESCC and nonmalignant surrounding mucosa. **(D)** Correlation between *FOXM1* and *PIK3R3* expressions in tumor samples by RT-qPCR. **(E)** Correlation between fold-change expression (ESCC/nonmalignant surrounding mucosa) of *FOXM1* and *PIK3R3*.

In order to better understand the role of FOXM1 overexpression in ESSCC carcinogenesis, we silence *FOXM1* (Figure 4A) in ESCC cells by transfecting with a specific siRNA targeting *FOXM1* (TE-1-siFOXM1) siRNA. Then, the global gene expression profile was assessed in the *FOXM1* silenced and control, scrambled (TE-1-SCR) siRNA, transfected cells. *FOXM1* silencing resulted in 222 DEG (Supplementary Table 4), comprising 127 overexpressed and 95 underexpressed genes. Among these latter, phosphoinositide-3-kinase regulatory subunit 3 (*PIK3R3*) was found to be 50% underexpressed in TE-1-siFOXM1 cells, a finding subsequently confirmed by RT-qPCR. Similarly, induction of *FOXM1* expression by TE-1 cells transfection with an expression vector resulted in a 2.2-fold increase in *PIK3R3* expression (Figure 4B). *PIK3R3* expression was subsequently assessed in 57 ESCC and NSM paired samples, showing a 3.4-fold overexpression in tumors (Figure 4C). Additionally, a positive correlation was found between *PIK3R3* and *FOXM1* expressions in ESCC samples (Figure 4D). Noteworthy, was also observed correlation in fold-changes expression levels of *PIK3R3* and *FOXM1* (Figure 4E).

**Figure 5:**
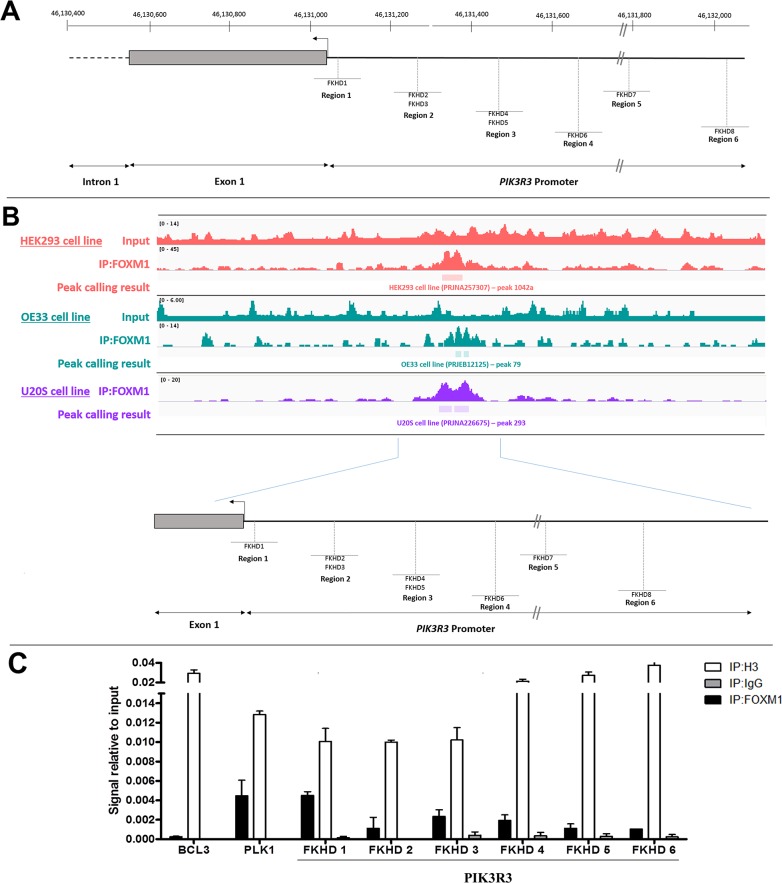
Binding of FOXM1 onto *PIK3R3* promoter region **(A)** Schematic representation of the position of putative the FOXM1 response elements (FKHD1–FKHD8) in the 5' regulatory region of *PIK3R3*. The interval 46,598,400 – 46,599,900 on Chr 1p is represented (reference: GRCh38. p2). The position of *PIK3R3* transcription initiation site and of exon 1 are shown. The proximal part of intron 1 is represented by a black hatched bar. The FKHDs analyzed are comprised in regions 1, 2 and 3 and are located about 10, 120 and 230 bases, respectively, upstream of the transcription initiation site. **(B)** Analyses of FOXM1 binding on 5' regulatory region of *PIK3R3* in different cell lines using ChIP-Seq data from SRA data-base. The ChIP-Seq binding patterns of FOXM1 in *PIK3R3* promoter sequence are compared in three cell lines, demonstrating common peaks. Peak calling result of FOXM1 ChIP-Seq in these three cell lines is demonstrated and show a significative peak on 5' regulatory region of *PIK3R3* (p<0.0001). **(C)** TE-1 cells was used for evaluation of FOXM1 binding to *PIK3R3* promoter. Cross-linked chromatin was immunoprecipitated with anti-FOXM1 antibody (IP:FOXM1) or with anti-histone H3 antibody (IP:Histone H3), used as a positive control, or with anti-normal rabbit IgG antibody (IP:IgG), used as a negative control. Following immunoprecipitation, purified DNA was analyzed by qPCR, using primers specific for regions of *PIK3R3* promoter region encompassing FKHD1-FKHD6. The amount of immunoprecipitated DNA in each sample is represented as a signal relative to the total amount of input chromatin (=1). BCL3 and PLK1 were used as negative and positive controls, respectively, for FOXM1 binding. Data presented in triplicate qPCR experiments ±SD.

Then, we investigated whether the correlation between FOXM1 and PIK3R3 expression might depend on the ability of FOXM1 to regulate PIK3R3 expression. Then, first we searched for potential FOXM1-binding sites (forkhead domain, FKHD) were searched in the *PIK3R3* promoter region. Eight FKHD were identified in six different regions (Figure 5A) and, furthermore, with three MYBL2 response elements at close FKHD sites (Supplementary Table 5). MYBL2 is a well-known cooperative transcriptional factor of FOXM1 [10].

Following identification of FKHD sites, the interaction between FOXM1 and the *PIK3R3* promoter was investigated. Analysis of FOXM1-ChIP-Seq data of three different cell lines, HEK293, OE33 and U20S showed statistically significant DNA peaks (p < 0.0001), corresponding to the *PIK3R3* promoter region, in all cell lines following FOXM1 immunopreciptation (Figure 5B). Moreover, ChIP assay performed in TE-1 cells showed that FOXM1 binds to all the FKHD evaluated within *PIK3R3* promoter region, confirming FOXM1-ChIP-Seq data analysis. The same result was observed for *PLK1* promoter region, used as FOXM1-binding positive control, since it is a known target of this transcriptional factor, whereas no FOXM1 binding onto *BCL3* promoter region, used as a negative control, was observed (Figure 5C).

**Figure 6:**
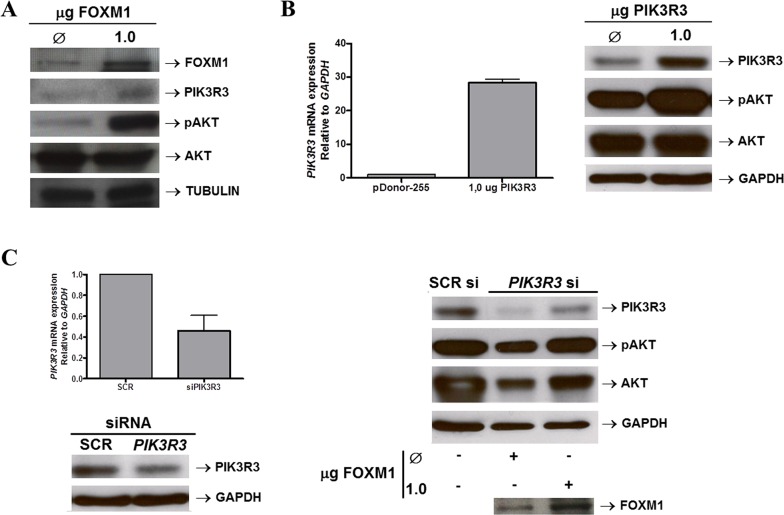
FOXM1 activates PIK3/AKT pathway by upregulating PIK3R3 **(A)** FOXM1 expression was induced in the ESCC-derived cell line TE-1 by transfection of 1μg of a FOXM1 expression vector (pcDNA3-FOXM1). Control TE-1 cells (Ø) were transfect with1μg of a control, empty vector (pcDNA3). Western blot analysis shows the induction of FOXM1, evaluated as a positive control, PIK3R3 and phospho-AKT levels upon FOXM1 overexpression. AKT expression was also evaluated and did not show significant changes after FOXM1 overexpression. Tubulin expression was evaluated as loading control. **(B)** PIK3R3 expression was induced in the ESCC-derived cell line TE-1 by transfection of 1μg of a PIK3R3 (pDonor255-PIK3R3). Control TE-1 cells (Ø) were transfect with1μg of a control, empty vector (pDonor255). RT-qPCR analysis show PIK3R3 induced mRNA expression following the PIK3R3 expression vector transfection in TE-1 cells (left panel). Western blot analysis (right panel) shows the induction of PIK3R3, evaluated as a positive control, PIK3R3 and phospho-AKT levels upon PIK3R3 overexpression. AKT expression was also evaluated and did not show significant changes after FOXM1 overexpression. GAPDH expression was evaluated as loading control. **(C)** TE-1 cells were silenced for*PIK3R3* by transfecting specific siRNA. Control cells were transfected with a non-specific siRNA sequence (SCRsi). Following *PIK3R3* silencing, TE-1 cells had FOXM1 levels induced by transfection of a FOXM1 expression vector (pcDNA3-FOXM1). Control TE-1 cells (Ø) were transfect with1μg of a control, empty vector (pcDNA3). (Left panel)RT-qPCR and Western blot analyses show that *PIK3R3* silencing was efficient and reduced both PIK3R3 mRNA and protein levels. (Right panel) PIK3R3, phospho-AKT and AKT levels were evaluated, by Western blot, in TE-1 *PIK3R3*-silenced cells and overexpressing FOXM1. The decrease in PIK3R3 levels was partially restored after FOXM1 overexpression and resulted in an increase of phospho-AKT levels, as well as a slight increase of those of AKT. GAPDH expression was evaluated as loading control.

The possibility that FOXM1 regulation of *PIK3R3* expression would activate the PI3K/AKT pathway was also investigated. FOXM1 overexpression in TE-1 cells was found capable of increasing PIK3R3 expression and the levels of phospho-AKT, the key effector of the PI3K/AKT pathway activity (Figure 6A). Furthermore, PIK3R3 overexpression in TE-1 cells also resulted in a remarkable increase of phospho-AKT levels, corroborating previous findings (Figure 6B). Consistently, *PIK3R3* silencing in TE-1 cells reduced PIK3R3 and, slightly, phospho-AKT levels. Moreover, *PIK3R3* silencing in TE-1 cells followed by induced *FOXM1* overexpression was capable of partially restoring both PIK3R3 and phospho-AKT levels, similarly to SCRsi controls (Figure 6C). Therefore, these results indicated that FOXM1-induced AKT phosphorylation might be mediated by PIK3R3 upregulation.

**Figure 7:**
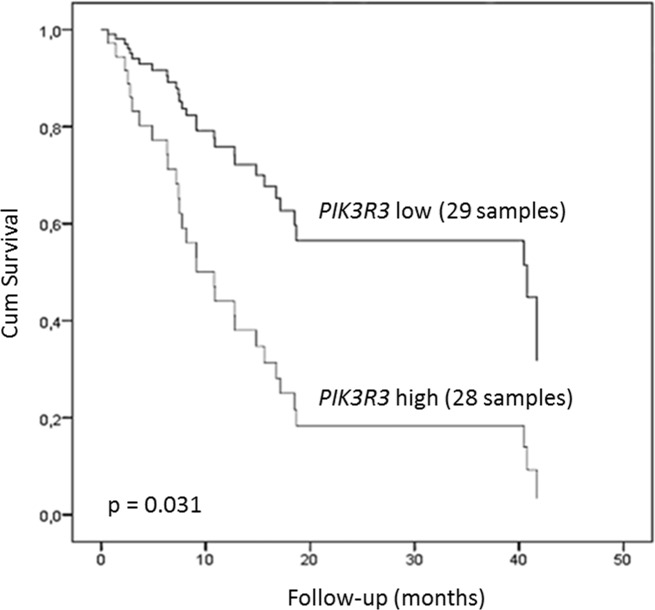
Clinical relevance of *FOXM1* and *PIK3R3* expression in ESCC Kaplan–Meier plot of ESCC patients overall survival. Patients were plotted according to *PIK3R3* expression level. High and low expression groups of patients were established according to *PIK3R3* tumor expression median value (0.010972226). Data are adjusted by age, stage, and *FOXM1* expression. ^*^ p < 0.05; ^***^ p < 0.0001; Abbreviations: r, Spearman correlation coefficient; CI, confidence interval.

Survival analysis, with respect to gene expression, after age and tumor stage adjustments, revealed that high *FOXM1* expression was associated with a poor ESCC patients prognosis, presenting a 2.73-fold increase of the risk of death (p = 0.03; 95% CI 1.11-5.93). In a similar way, *PIK3R3* expression was also related to worse prognosis, with a 2.69-fold increase of the risk of death (p = 0.026, 95% CI 1.20-7.27). Finally, the Cox's Regression Models including both genes showed that survival was associated only to *PIK3R3* expression (p = 0.031, 95% CI 1.09-6.79), patients with high *PIK3R3* expression showed a 2-fold lower survival time than those with low *PIK3R3* expression (9.13 months versus 18.5 months) (Figure 7). On the other hand, expression levels of *PLK1* (p = 0.99), *ETV5* (p = 0.83), *CDK1* (p = 0.36), *MMP9* (p = 0.92), *MMP12* (p = 0.39), and *CCNB1* (p = 0.11) were found to be unrelated to outcome.

## DISCUSSION

In this study, FOXM1 was shown to play a crucial role in modulating the expression of several genes in ESCC. A novel FOXM1 target, *PIK3R3*, was identified, and its overexpression was not only shown to mediate FOXM1-activation of the PI3K/AKT pathway, but also to confer poor prognosis to ESCC patients.

The ESCC global gene expression profile identified 1,328 differentially expressed genes in ESCC compared to their counterparts in paired NSM samples. Paired sample size above 12 did not provide additional significant information on DEG number, suggesting that 15 paired samples were adequate for identifying the most common differentially expressed genes in ESCC.

Master regulator and pathway enrichment analyses identified FOXM1 as a central transcriptional factor responsible for DEG in ESCC. *FOXM1* as well as some of its regulated genes, like *CCNB1*, *CDK1*, *PLK1*, *ETV5*, *MMP9,* and *MMP12*, were found to be overexpressed in ESCC and their expression was positively correlated. FOXM1 overexpression has also been reported in several tumors, such as hepatocellular, pancreatic, colorectal and laryngeal cancer [11–14], with involvement in different mechanisms of oncogenesis, like cell proliferation, invasion, angiogenesis, and metastasis [15–19]. Previous reports on FOXM1 expression in ESCC showed that low levels of cytoplasmic FOXM1 were associated with the initial phase of esophageal carcinogenesis [20] while its nuclear expression negatively affected patient survival [21]. Furthermore, increased FOXM1 expression through deregulation of miR204 has been shown to contribute to the epithelial-mesenchymal transition of ESCC cells and to be associated with ESCC invasion [22]. Among FOXM1 targets, CCNB1 and PLK1 levels have been described as prognostic biomarkers for ESCC [23, 24], whereas MMP12 expression was shown to be associated with ESCC progression [25]. In esophageal adenocarcinoma, changes in the expression of network genes regulated by FOXM1 have been useful in predicting prognosis [26]. Altogether, with the findings herein reported, these studies suggest that FOXM1 plays a central role as a transcriptional factor in esophageal carcinogenesis.

Comparisons of gene expression profiles of *FOXM1* silenced and control TE-1 cells were successful in identifying FOXM1 targets in ESCC by microarray analysis. One of these DEG, a novel potential *FOXM1* target, *PIK3R3* was underexpressed following *FOXM1* abrogation in TE-1 cells, while *PIK3R3* was found to be overexpressed and positively correlated with *FOXM1* expression in ESCC samples.

Reanalysis of open access ChIP-Seq data on FOXM1 immunoprecipitation showed that FOXM1 binds to a region containing FKHD elements in the *PIK3R3* promoter in different cell lines. Moreover, ChIP assay performed in TE-1 cells showed that FOXM1 directly interacts with its consensus regions within *PIK3R3* promoter. Specifically, FOXM1 binding to FKHD 1 region, harbored in *PIK3R3* promoter, shows similar levels to that presented in *PLK1* promoter, reinforcing the hypothesis that *PIK3R3* is transcriptionally regulated by FOXM1 in ESCC. These findings were coincident with previous reports on the binding of the FOXM1conserved domain with FKHD motif (RYAAAYA) DNA regions [27].

PIK3R3 (p55γ subunit) is a regulatory subunit forming heterodimers with class IA p110α catalytic subunit (PIK3CA) [28], the most studied catalytic subunit in cancer [29]. Compared with other regulatory subunits encoded by *PIK3R1* and *PIK3R2*, p85α and p85β respectively, PIK3R3 contains a unique NH2 terminal region that triggers specific functions mediated by binding to key cell growth proteins, including pRB1 (retinoblastoma protein) and PCNA (proliferating cell nuclear antigen) [30]. *PIK3R3* overexpression has been shown to play a role in colon, liver, and pancreatic tumors by promoting proliferation, migration and invasion across the epithelial-mesenchymal transition [31].

Induced *FOXM1* overexpression in TE-1 cells led to increased levels of both PIK3R3 and phospho-AKT, while increased phospho-AKT levels were also detected with *PIK3R3* overexpression. Additionally, FOXM1 overexpression in *PIK3R3*-silenced TE-1 cells partially restored PIK3R3 levels and, consequently, those of phospho-AKT. These findings lead us to propose that FOXM1 overexpression induced activation of the AKT/PI3K pathway by upregulating PIK3R3 expression.

PI3K signaling is activated in human cancers by several different mechanisms, including mutations or amplification of genes that encode key components of the PI3K pathway, like *PIK3CA* and *AKT1*, or loss of *PTEN* [32]. *PIK3CA* and *PTEN* non-concurrent mutations have been shown to occur in 13% and 9% of ESCC, respectively, while *PIK3CA* amplification has not been observed in ESCC [33]. Other studies have reported the presence of *PIK3CA* mutations in 2 -17% of the ESCC samples [34–36]. However, *PIK3CA* overexpression or *PIK3R1* and *PTEN* underexpression were not herein detected by microarray analysis. These findings further supported our proposition that *PIK3R3* overexpression, induced by FOXM1, is a mechanism for activation of the PI3K/AKT pathway in ESCC.

Interestingly, it has been shown that the phosphorylation cascade triggered by PI3K/AKT may indirectly result in FOXM1 activation by inhibiting FOXO3. FOXO3 is a transcriptional antagonist of FOXM1, acting in three different ways: (i) displacing FOXM1 from the FKHDs of target genes, (ii) transcriptionally silencing FOXM1 and (iii) recruiting chromatin-remodeling proteins, which promote chromatin condensation and limit the access of transcription factors, such as FOXM1. In this way, activation of the PIK3/AKT signaling pathway inhibits FOXO3 and indirectly induces FOXM1 activity [19]. Together with our data, these observations suggest an intricate positive feedback between FOXM1 and PI3K/AKT pathway, which may contribute to ESCC carcinogenesis.

The high expression of *FOXM1* and *PIK3R3* was associated with a poor overall survival rate, although multivariate analysis showed that *PIK3R3* expression was classified an independent prognostic variable. This observation might indicate that previous results associating FOXM1 expression with a worse prognosis of ESCC patients may do so through the induction of *PIK3R3* [33, 34]. Altogether, these results suggest that inhibition of the PI3K/AKT pathway might be a useful strategy for targeted treatment of ESCC patients. At present, phase II studies using the pan-PI3K inhibitor BKM120 with ESCC patients are underway [37].

We conclude that FOXM1 seems to play a central role in determining gene expression profile of ESCC, and stimulates the PI3K/AKT pathway by inducing *PIK3R3* expression, which is associated with a worse prognosis in ESCC patients. Further work in independent cohorts is required for validating its role as a prognostic biomarker and functional studies are needed for demonstrating *PIK3R3* contribution to esophageal carcinogenesis.

## MATERIALS AND METHODS

### Paired samples of tumor and nonmalignant surrounding mucosa

A total of 57 paired biopsies of ESCC and nonmalignant surrounding mucosa (NSM; histopatologically normal mucosa, 3 to 5 cm from tumor borders) were collected between 2006 and 2014 by the Endoscopy Service of the Instituto Nacional de Câncer (Rio de Janeiro, Brazil). Histopathological profiling was performed by the Pathology Department. At time of collection, patients had not undergone chemotherapy or radiotherapy. Samples were separated into two groups: an investigation set (15 pairs of ESCC and NSM) and a validation set (42 pairs), while all samples were used for overall survival analysis.

Patients signed an informed consent allowing the use of samples and clinicopathologic data (Supplementary Table 6), and the project was approved by the institutional Ethics Committee.

### ESCC gene expression profiling

Total RNA was isolated from frozen biopsies with RNeasy Mini Kit (Qiagen, Inc.) and cDNA was synthesized with WT Expression Kit, biotinylated, and applied to genechip Human Exon 1.0 ST array (Affymetrix, Inc.). cDNA samples from the same patients were processed in the same batch. The raw data were normalized in Expression Console software (Affymetrix) using the robust multi-array average (RMA) method. Subsequent analysis of gene expression data was carried out with the freely available statistical computing language R using the Limma package available from the Bioconductor project to obtain quantitative expression levels for coding genes. Differentially expressed genes (DEG) were identified by the following criteria: p < 0.05, fold-change expression cutoff |2.0|, and linear step-up false discovery ratio with p ≤ 0.05. Microarray data are available at GEO Accession Browser (accession number GSE75241 and GSE75243) [38–40].

### Enrichment and master regulator analyses of differentially expressed genes

Post-processing analyses of DEG were performed with Gene Ontology and KEGG database using WEB-based gene set analysis toolkit [41]. A hypergeometric test was used for enrichment evaluation analysis, with statistical parameter p < 0.001, following Benjamini & Hochberg for multiple test adjustment.

The ARACNE algorithm was used for identifying the master regulators of the ESCC expression profile. This algorithm relies on expression data for calculating pairwise Mutual Information (MI). These findings were used to build an interaction model between the differentially expressed transcription factors (TF) and DEG, looking forward to identifying whether TF activation might be responsible for the transition from surrounding mucosa to tumor. The transcriptional network was built with the RTN package [42], generating a degree of statistical dependency between these variables [43]. Master Regulator Analysis (MRA) identified DEG in each regulon with Fisher's exact test, indicating transcription factors that might be operating as master regulators of the observed gene expression profile (p < 0.05) [44].

### DNA microarray validation by quantitative PCR

Thirteen DEG and one gene without differential expression were selected for validation by RT-qPCR (Supplementary Table 7). cDNA was synthesized with SuperScript^TM^ II Reverse Transcriptase (Invitrogen^®^) and quantitative PCR was carried out with Quantifast SYBR Green PCR kit (Qiagen) in an RG 6000 thermal cycler (Qiagen). Gene expression was normalized with respect to *GAPDH* expression, and relative quantification was estimated by the ΔCt method [45].

### Cell line and treatments

The ESCC cell line TE-1 was used for *in vitro* analyses. The TE-1 cell line was kindly provided by Dr. Pierre Hainaut (IARC, France). Cells were authenticated using Powerplex 18D STR System (Promega) and were routinely tested for Mycoplasma using Mycosensor PCR assay kit (Agilent). *FOXM1* and *PIK3R3* expression was silenced in separate assays using 2×10^5^ cells/ well / 2 mL, twice transfected into 6-well plates, at one 24 hour interval, one with specific siRNA targeting *FOXM1* (sc-43769, Santa Cruz, 1 μl of a 20 μM solution), and another targeting *PIK3R3* (AM16708, Ambion, 1μl of a 20μM solution); each one with a negative siRNA control (siRNA control A, sc-37007, Santa Cruz, or SCR siRNA, #4611, Ambion) using Lipofectamine 2000 (Invitrogen). Cells were harvested 24 h following the second transfection. *FOXM1* and *PIK3R3* overexpression was induced using the same amount of TE-1 cells, separately transfected into 6-well plates with increasing amounts of pcDNA3-FOXM1 [46], PIK3R3 (#70460, Addgene) expression vectors, and an empty vector (pcDNA3, Invitrogen, or pDonor255, Gateway^®^ pDONR™ Vectors, Invitrogen) for negative controls. Cells were harvested 24 hours after transfection. Following *PIK3R3* silencing, cells were transfected for *FOXM1* overexpression, using a transfection with the empty vector as control, 6 hours after the second *PIK3R3* silencing transfection. Twenty four hours later, cells were collected.

DNA microarray analysis was carried out with the ESCC-derived cell line, TE-1, with and without *FOXM1* silencing, each with two replicates. A 1.5-fold, change-in-expression cutoff was used for identifying differentially expressed genes following *FOXM1* silencing.

ChIP assay was performed in TE-1 cells. Firstly, 1% formaldehyde was added to culture medium for 10 min at room temperature to cross-link DNA and proteins, followed by neutralization by addition of glycine (pH 2.5). Cells lysis, immunopreciptation and DNA isolation were performed with SimpleChIP^®^ Enzymatic Chromatin IP Kit (Magnetic Beads) according to the manufacturer's protocol (Cell Signaling). Lysates were sonicated and FOXM1 immunopreciptation was performed with 9 μg of FOXM1 antibody (FOXM1 (C-20): sc-502, Santa Cruz). Immunopreciptation with histone H3 and IgG antibodies was used as positive and negative controls, respectively (Cell Signaling). Quantitative PCR with specific primers for forked head domain regions (FKHD) was carried out for evaluating the amount of immunopreciptated DNA, as well as for *PLK1* and *BCL3* promoters regions, positive and negative FOXM1-binding controls, respectively (Supplementary Table 2).

Western Blotting analyses was carried out following protein extraction from cells with RIPA-like buffer (250 mM NaCl, 50 mM TRIS-HCl pH 7.4, 0.1% SDS, 2 mM DTT and 0.5% NP-40) containing protease inhibitors (Complete-Mini, Roche). Protein concentration was determined by Bradford assay (Bio-Rad). Standard curve was estimated with bovine serum albumin (BSA) and 50 μg of total protein extract in 8.0% SDS PAGE (PAGE). Proteins were transferred to a nitrocellulose membrane (Roche) and probed with appropriate antibodies for 1 hour. Anti-FOXM-1 (sc-502, Santa Cruz) and anti-PIK3R3 (ab186612, Abcam) were used at 1:250 and 1:100 dilutions, respectively, and anti-pAKT (4060, Cell Signaling), anti-pan AKT (SAB4300259, Sigma) and anti-GAPDH (sc-32233, Santa Cruz) primary antibodies were used at 1:1,000 dilutions. Membranes were subsequently incubated with horseradish peroxidase-conjugated secondary antibody (1:10,000) for 1 hour, and detection was performed with enhanced chemiluminescence (ECL Kit, Amersham).

### Statistical and *in silico* analyses

Differences in gene expression between ESCC and NSM were evaluated with RT-qPCR, paired t test or Wilcoxon matched pair test. Pearson or Spearman's rank correlation was used for assessing whether the expression of different genes were correlated using GraphPad Prism 5 software. Univariate survival was estimated by the Kaplan–Meier method and log-rank test. Variables with p < 0.2 were selected for multivariate analysis. Finally, Cox regression was applied with the stepwise forward method. For best coverage of important variables, we included those with statistical and clinical significance on outcome, like age and tumor stage. SPSS 20.0 software was used for survival analyses.

MatInspector software was used for identifying FOXM1-binding sites (FKHD) in the 5'sequence of *PIK3R3* coding region (sequences GXP_260387-PIK3R3/Human, GXP_1817525-PIK3R3/Human, and GXP_4400643-PIK3R3/Human) [47]. Sequences derived from FOXM1 ChIPp-Seq experiments using three cell lines: HEK293, OE33, and U20S, derived from embryonic normal kidney, esophageal adenocarcinoma and osteosarcoma, respectively (NCBI Bioprojects accession numbers PRJNA257307, PRJEB12125 and PRJNA226675), were downloaded from SRA database and analyzed with SraToolkit, FASTQc, bowtie toll and IGV toll [48–50].

## SUPPLEMENTARY MATERIALS FIGURE AND TABLES








