# Supplementary material for: Esophageal squamous cell carcinoma transcriptome reveals the effect of FOXM1 on patient outcome through novel PIK3R3 mediated activation of PI3K signaling pathway
Source: Oncotarget. 2018 Mar 30;9(24):16634–47. doi: 10.18632/oncotarget.24621 (PMC5908275; doi:10.18632/oncotarget.24621)
Supplement: Supplementary file 1 [file oncotarget-09-16634-s001.pdf]

Esophageal squamous cell carcinoma transcriptome reveals the effect of *FOXM1* on patient outcome through novel PIK3R3 mediated activation of PI3K signaling pathway

SUPPLEMENTARY MATERIALS

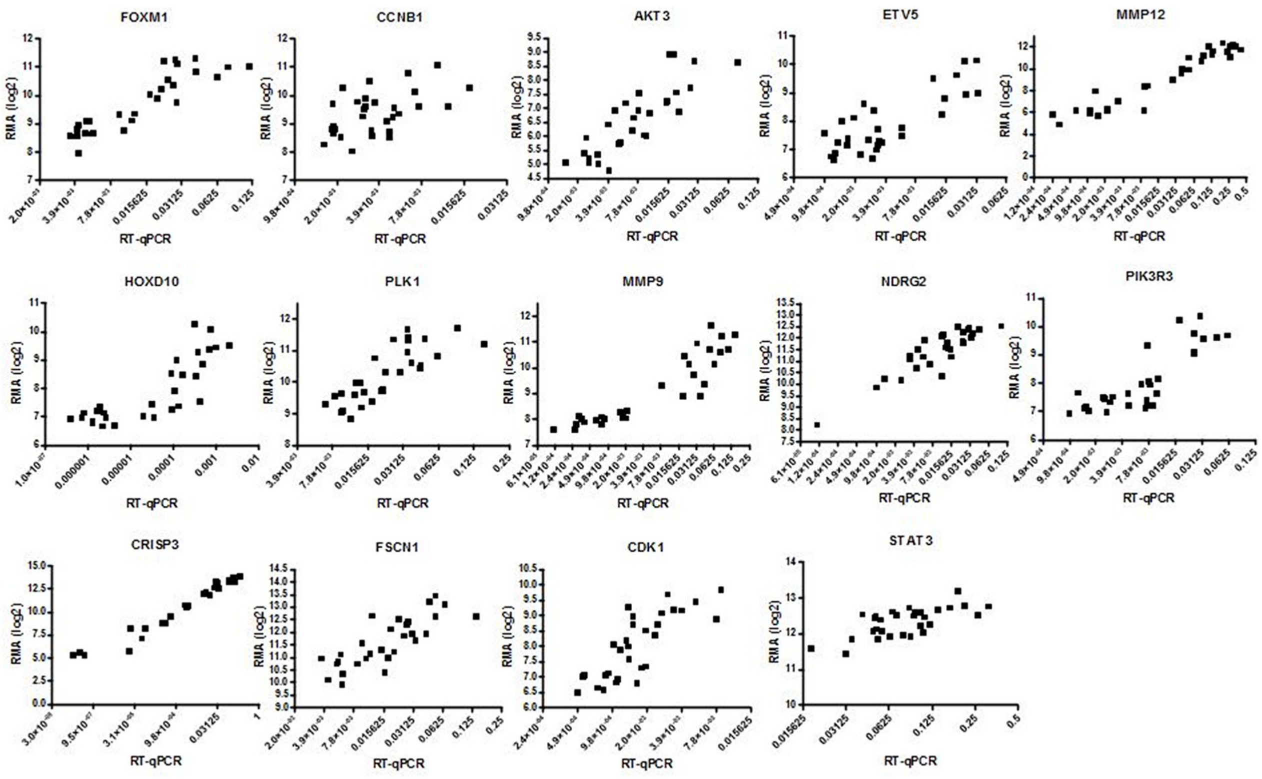

Supplementary Figure 1: Scatter plots of correlation between DNA microarray and RT-qPCR. Microarray values are in log2 scale, whereas RT-qPCR gene expression values are relative to *GAPDH*. All individual genes showed a significant p value ($p < 0.05$). The correlation coefficients for each gene were as follows: *FOXM1*, r = 0.89; *CCNB1*, r = 0.44; *AKT3*, r = 0.66; *ETV5*, r = 0.68; *MMP12*, r = 0.93; *HOXD10*, r = 0,83; *PLK1*, r = 0.83; *MMP9*, r = 0.92; *NDRG2*, r = 0.83; *PIK3R3*, r = 0.78; *CRISP3*, r = 0.96; *FSCN1*, r = 0.84; *CDK1*, r = 0.79; *STAT3*, r = 0.64.

Supplementary Table 1: Differentially expressed genes (DEG) between ESCC and nonmalignant surrounding mucosa identified by gene expression DNA microarray. DEG are sorted according to the absolute variation in expression. Fold-change is based on log2. NA, gene symbol corresponding to un-annotated probe.

See Supplementary File 1

Supplementary Table 2: Major regulator gene outcome of transcriptional network analysis. The 176 transcriptional factors described as responsible for ESCC gene expression profile are sorted by adjusted p value.

See Supplementary File 2

Supplementary Table 3: Matrix of correlation analyses between *FOXM1* and its pathway members

	Tumor Gene Expression						
	**FOXM1**	**ETV5**	**PLK1**	**CDK1**	**MMP9**	**MMP12**	**CCNB1**
**FOXM1**		0,217	0,471	0,469	0,081	0,153	0,103
**ETV5**	0,1043		0,281	0,351	0,364	0,307	0,272
**PLK1**	0,0002	0,035		0,757	0,350	0,473	0,419
**CDK1**	0,0002	0,008	9,2E-12		0,383	0,397	0,366
**MMP9**	0,5486	0,005	7,6E-03	0,003		0,713	0,397
**MMP12**	0,2568	0,020	2,0E-04	0,002	4,8E-10		0,368
**CCNB1**	0,4444	0,041	1,2E-03	0,005	2,2E-03	0,005	
	**Expression Fold Change**						
	**FOXM1**	**ETV5**	**PLK1**	**CDK1**	**MMP9**	**MMP12**	**CCNB1**
**FOXM1**		0,363	0,600	0,611	0,304	0,398	0,435
**ETV5**	0,005		0,314	0,346	0,474	0,383	0,273
**PLK1**	0,000	0,017		0,688	0,404	0,455	0,707
**CDK1**	0,000	0,008	3,3E-09		0,314	0,328	0,597
**MMP9**	0,022	0,000	1,8E-03	0,017		0,695	0,342
**MMP12**	0,002	0,003	3,8E-04	0,013	2,0E-09		0,540
**CCNB1**	0,001	0,040	7,9E-10	0,000	9,3E-03	1,4E-05	

This file has two matrices, the first one describing correlation analysis of gene expression in tumors and the second one showing data for fold-change in expression. Black values, correlation coefficients; Red values, p values.

Supplementary Table 4: Differentially expressed genes (DEG) after *FOXM1* silencing in ESCC-derived TE-1 cell line. DEG are sorted by absolute fold change (linear) values. Only well-annotated probe sets were categorized as differently expressed.

See Supplementary File 3

Supplementary Table 5: *In silico* analysis of the *PIK3R3* promoter using MatInspector software resulted in eight putative elements responsible for the effects of FOXM1

MatInspector sequence			FKHD				MYBL2			
	FOXM1 response element	Region #	Start position	End position	Anchor position	Strand	Start position	End position	Anchor position	Strand
GXP_260387(PIK3R3/human)	3	2	−119	−135	−127	−				
GXP_260387(PIK3R3/human)	6	4	−323	−339	−331	+	−470	−490	−480	+
GXP_1817525(PIK3R3/human)	1	1	−2	−18	−10	−				
GXP_1817525(PIK3R3/human)	2	2	−115	−131	−123	+				
GXP_1817525(PIK3R3/human)	4	3	−220	−236	−228	+				
GXP_1817525(PIK3R3/human)	5	3	−224	−240	−232	−	−225	−245	−235	−
GXP_4400643(PIK3R3/human)	7	5	−1060	−1076	−1068	−	−1092	−1112	−1102	−
GXP_4400643(PIK3R3/human)	8	6	−1198	−1214	−1206	+				

The promoter regions are designated according to the position before the transcription initiation codon.

Supplementary Table 6: Characteristics of the individuals included in this study

**Gender**	**Male**	44	(77.19%)
	**Female**	13	(22.81%)
**Age (years)**	**Median**	59.33	
	**Range**	39 - 79	
**Follow-up (months)**	**Median**	11.87	
	**Range**	0.63 - 85.9	
**Tabacco Smoking**	**No**	6	(12.77%)
	**Yes**	41	(87.23%)
	**NA**	10	
**Alcohol Drinking**	**No**	7	(14.89%)
	**Yes**	40	(85.11%)
	**NA**	10	
**ESCC Localization**	**Upper**	15	(26.32%)
	**Middle**	33	(57.89%)
	**Lower**	8	(14.04%)
	**Middle - Lower**	1	(1.75%)
**Tumor Differentiation**	**Well**	2	(3.51%)
	**Moderate**	42	(73.68%)
	**Poor**	13	(22.81%)
**Tumor Stage**	**I**	2	(4.35%)
	**II**	13	(28.26%)
	**III**	20	(43.48%)
	**IV**	11	(23.91%)
	**NA**	11	

[*] Number of patients may vary due to missing data (NA).

Supplementary Table 7: Sequences of primers enrolled in the study

Target		Primer Sequence	Target ID Sequence	Amplicon Size (bases)
AKT3	F	5’ CACCACCTGAAAAATGTCAGCA 3’	NM_181690.2	113
	R	5’ AAGCCAACAGTTCCATGGGT 3’		
PIK3R3	F	5’ TATATCTCCTGGCGACCTGC 3’	NM_003629.3	154
	R	5’ GCTTTGGTGGAAGAGCTGGA 3’		
CDK1	F	5’ AAACTGCTCGCACTTGGCTT 3’	NM_001786.4	115
	R	5’ GGTATGGTAGATCCCGGCTTAT 3’		
PLK1	F	5’ AGTACGGCCTTGGGTATCAG 3’	NM_005030.5	118
	R	5’ GTGCCGTCACGCTCTATGTA 3’		
STAT3	F	5’ AGAAACAGTTGGGACCCCTG 3’	NM_139276.2	119
	R	5’ GCTCCATTGGGAAGCTGTCA 3’		
MMP12	F	5’ GACTACACATTCAGGAGGCACA 3’	NM_002426.4	164
	R	5’ TGCCACGTATGTCATCAGCA 3’		
MMP9	F	5’ GCGCTGGGCTTAGATCATTC 3’	NM_004994.2	130
	R	5’ GTTCAGGGCGAGGACCATAG 3’		
FSCN1	F	5’ GCCTCCAGCAAGAATGCCAG 3’	NM_003088.3	179
	R	5’ ATGATGGGGCGGTTGATGAG 3’		
GUSB	F	5’ TCGATGACATCACCGTCACC 3’	NM_000181.3	139
	R	5’ GTCCCATTCGCCACGACTTT 3’		
FOXM1	F	5’ AACCTTTCCCTGCACGACAT 3’	NM_202002.2	124
	R	5’ GGTCCAGTGGCTTAAACACC 3’		
HOXD10	F	5’ CAGGAGAAGGAAAGCAAAGAGGA 3’	NM_002148.3	112
	R	5’ CCAGCGTTTGGTGCTTAGTG 3’		
GAPDH	F	5’ TGTGAACGGATTTGGCCGTA 3’	NM_002046.5	220
	R	5’ TCGCTCCTGGAAGATGGTGA 3’		
CCNB1	F	5’ GTAATGTTGTAGAGTTGGTGTCC 3’	NM_031966.3	100
	R	5’ CATGGTGCACTTTCCTCCTT ‘3		
ETV5	F	5’ ACTGGAAGGCAAAGTCAAAC 3’	NM_004454.2	114
	R	5’ GCTGGGTCATCAAGAAGGGTGA 3’		
NDRG2	F	5’ GCAAAGAGAGGAGAGACCCC 3’	NM_201535.1	74
	R	5’ TATTGGCTGGATGCAGTGGG 3’		
CRISP3	F	5’ TCTGGAAACCACTGCAATGAC 3’	NM_006061.2	106
	R	5’ AGCAGTAAAAGCGGGATCCTT 3’		
FKHD Region 1	F	5’ AGGAGCAAGGCAGACAAAAG 3’	NC_000001.11	85
	R	5’ GAGGGAAGAGAGGCAAGGTG 3’		
FKHD Region 2	F	5’ TCTTTTCCCCTTCTGCCTTT 3’	NC_000001.11	91
	R	5’ GGCCAATCAGGAGACAGC 3’		
FKHD Region 3	F	5’ GACCAAGCCTCAGCAGGA 3’	NC_000001.11	99
	R	5’ AAAGGAAGAAAACGCAAACG 3’		
FKHD Region 4	F	5’ CTCTGTGTCCGCTCCCTTC 3’	NC_000001.11	94
	R	5’ CCCGGACGAGTAAGGAGA 3’		
FKHD Region 5	F	5’ GGAAATGGAGCAGTTTTTGG 3’	NC_000001.11	85
	R	5’ TGTGTCTCCTGAAGATTGTGC 3’		
FKHD Region 6	F	5’ AAACGTGGCAACTCTTCCAT 3’	NC_000001.11	81
	R	5’ GCCTGATTTAAGCCCTAGCA 3’		
PLK1	F	5’ GGGCGGGTTTGGATTTTA 3’	NC_000016.10	164
	R	5’ AGTCACTGCAGCACTCATGC 3’		
BCL3	F	5’ GAGAAACTAAGACCTTCCCTC 3’	NC_000019.10	99
	R	5’ AGCTGCACCATGCTAAGGCT 3’		
