# Supplementary material for: Esophageal squamous cell carcinoma transcriptome reveals the effect of FOXM1 on patient outcome through novel PIK3R3 mediated activation of PI3K signaling pathway
Source: Oncotarget. 2018 Mar 30;9(24):16634–47. doi: 10.18632/oncotarget.24621 (PMC5908275; doi:10.18632/oncotarget.24621)
Supplement: Supplementary file 2 [file oncotarget-09-16634-s002.doc]

Supplementary Table 1: Differentially expressed genes (DEG) between ESCC and nonmalignant surrounding mucosa identified by gene expression DNA microarray.

| **Gene Symbol** | **Expression Variation - ESCC / Adj Mucosa (logFC)** | **Adjusted p value** |
| --- | --- | --- |
| MMP1 | 5,688 | 9,99E-13 |
| MMP12 | 4,820 | 5,71E-12 |
| NA | 4,628 | 1,11E-13 |
| LUM | 4,378 | 3,03E-12 |
| IL8 | 3,868 | 4,84E-10 |
| CCL18 | 3,861 | 5,76E-09 |
| GREM1 | 3,735 | 8,15E-09 |
| SPP1 | 3,678 | 1,23E-07 |
| IGJ | 3,626 | 8,22E-08 |
| COL12A1 | 3,618 | 1,39E-09 |
| LAMC2 | 3,617 | 5,71E-12 |
| MMP3 | 3,552 | 1,87E-08 |
| SEMA3C | 3,473 | 7,56E-11 |
| FN1 | 3,419 | 1,87E-07 |
| IFI44L | 3,372 | 2,72E-10 |
| PLA2G7 | 3,330 | 2,74E-14 |
| TGFBI | 3,195 | 4,15E-10 |
| POSTN | 3,109 | 5,06E-06 |
| FCGR2A | 3,098 | 7,56E-11 |
| MMP10 | 3,086 | 1,44E-08 |
| C1S | 3,080 | 2,46E-09 |
| CXCL1 | 3,044 | 9,82E-10 |
| CXCL10 | 3,043 | 1,66E-05 |
| MMP2 | 3,008 | 4,68E-10 |
| APOBEC3A | 2,930 | 2,74E-06 |
| IFI6 | 2,929 | 4,83E-09 |
| CXCL13 | 2,920 | 3,36E-06 |
| ODC1 | 2,830 | 3,40E-09 |
| SERPINE1 | 2,809 | 5,20E-07 |
| C3 | 2,795 | 5,89E-09 |
| PTGS2 | 2,790 | 1,50E-10 |
| ANO1 | 2,786 | 1,03E-06 |
| CST1 | 2,758 | 5,56E-05 |
| SLCO1B3 | 2,758 | 9,52E-05 |
| APOBEC3B | 2,757 | 1,84E-08 |
| TNFAIP6 | 2,747 | 4,83E-09 |
| IFIT3 | 2,724 | 9,33E-09 |
| PMP22 | 2,723 | 1,27E-11 |
| HAS2 | 2,722 | 2,63E-06 |
| ITGA5 | 2,693 | 1,78E-10 |
| FCER1G | 2,681 | 2,26E-12 |
| VCAN | 2,681 | 2,96E-08 |
| MLF1 | 2,678 | 9,56E-08 |
| TREM1 | 2,663 | 1,07E-07 |
| S100A7A | 2,648 | 8,92E-06 |
| THBS1 | 2,647 | 7,46E-09 |
| INHBA | 2,644 | 3,23E-06 |
| FCGR3A | 2,634 | 9,16E-11 |
| FADS1 | 2,624 | 4,06E-09 |
| NT5E | 2,620 | 3,13E-07 |
| IGFBP7 | 2,616 | 8,53E-13 |
| RSAD2 | 2,613 | 4,31E-07 |
| CTSK | 2,610 | 4,83E-09 |
| CTSL | 2,593 | 1,52E-09 |
| ASPN | 2,592 | 7,70E-07 |
| DDX60 | 2,564 | 8,26E-09 |
| IL1B | 2,547 | 5,05E-08 |
| CDH11 | 2,539 | 1,49E-07 |
| LOC440173 | 2,527 | 6,09E-08 |
| IGFBP3 | 2,526 | 4,11E-09 |
| IFIT1 | 2,523 | 2,43E-07 |
| NA | 2,519 | 8,39E-08 |
| PHLDB2 | 2,516 | 9,70E-08 |
| IL36G | 2,506 | 9,31E-05 |
| SPARC | 2,503 | 2,19E-09 |
| SLC16A1 | 2,473 | 1,26E-10 |
| PECAM1 | 2,470 | 7,50E-10 |
| CYP24A1 | 2,469 | 3,50E-05 |
| CCNA1 | 2,466 | 6,66E-05 |
| MET | 2,463 | 4,17E-11 |
| PMEPA1 | 2,461 | 6,93E-09 |
| FNDC3B | 2,454 | 3,03E-12 |
| MMD | 2,452 | 1,47E-09 |
| PLAUR | 2,439 | 2,84E-11 |
| BAG2 | 2,433 | 7,61E-09 |
| AQP9 | 2,423 | 5,08E-07 |
| CCR1 | 2,419 | 3,05E-10 |
| CXCL9 | 2,414 | 4,33E-05 |
| RNASE1 | 2,410 | 6,60E-10 |
| FST | 2,395 | 1,14E-05 |
| MMP9 | 2,395 | 3,06E-10 |
| KRT17 | 2,390 | 4,93E-08 |
| PRTFDC1 | 2,363 | 3,42E-07 |
| STC1 | 2,352 | 2,28E-09 |
| BAMBI | 2,344 | 1,15E-06 |
| FARP1 | 2,339 | 1,72E-08 |
| POPDC3 | 2,338 | 1,34E-07 |
| BST2 | 2,324 | 8,77E-06 |
| EIF5A2 | 2,322 | 2,79E-09 |
| BCAT1 | 2,316 | 6,17E-09 |
| COL1A2 | 2,304 | 9,92E-08 |
| APOL1 | 2,300 | 3,72E-07 |
| SERPING1 | 2,296 | 7,88E-09 |
| ANTXR2 | 2,293 | 1,18E-09 |
| IDO1 | 2,293 | 1,51E-06 |
| TGM2 | 2,287 | 8,41E-10 |
| DAB2 | 2,285 | 1,80E-10 |
| ACOT9 | 2,283 | 4,70E-10 |
| COL1A1 | 2,280 | 1,17E-07 |
| SERPINA1 | 2,280 | 8,91E-09 |
| LRP12 | 2,276 | 6,60E-09 |
| ECT2 | 2,272 | 4,06E-09 |
| IL2RA | 2,266 | 2,62E-09 |
| AIM2 | 2,232 | 1,22E-06 |
| EPCAM | 2,227 | 5,49E-07 |
| SEL1L3 | 2,220 | 8,41E-10 |
| ALCAM | 2,219 | 2,19E-06 |
| C3AR1 | 2,215 | 8,29E-10 |
| ISLR | 2,210 | 1,66E-07 |
| PLAT | 2,193 | 3,51E-06 |
| DPYSL3 | 2,192 | 6,21E-09 |
| LGALS1 | 2,175 | 1,04E-08 |
| CDC42EP3 | 2,173 | 1,06E-08 |
| THY1 | 2,170 | 3,46E-10 |
| LTBP1 | 2,169 | 5,17E-08 |
| ITGA6 | 2,165 | 2,08E-10 |
| C1R | 2,165 | 2,68E-08 |
| LY96 | 2,164 | 1,96E-08 |
| TNC | 2,163 | 1,16E-06 |
| PLAU | 2,145 | 4,83E-09 |
| FPR3 | 2,142 | 4,48E-09 |
| RPL39L | 2,141 | 2,84E-08 |
| RHOBTB3 | 2,138 | 8,95E-09 |
| TRAM2 | 2,131 | 2,14E-10 |
| TIMP1 | 2,125 | 1,24E-09 |
| ACTA2 | 2,123 | 1,58E-06 |
| COL3A1 | 2,122 | 1,67E-07 |
| MMP13 | 2,115 | 2,91E-06 |
| OSMR | 2,115 | 1,16E-08 |
| IFI44 | 2,112 | 1,04E-07 |
| HEY1 | 2,109 | 2,37E-06 |
| CDC6 | 2,107 | 2,81E-09 |
| SERPINH1 | 2,103 | 7,56E-11 |
| MAP1B | 2,102 | 1,41E-05 |
| DTL | 2,097 | 7,33E-10 |
| AKT3 | 2,096 | 6,97E-07 |
| TDO2 | 2,094 | 2,93E-08 |
| ABCA13 | 2,093 | 9,36E-05 |
| IL7R | 2,090 | 8,33E-07 |
| RFC4 | 2,087 | 1,52E-10 |
| ST6GAL1 | 2,087 | 2,14E-07 |
| NID1 | 2,080 | 4,83E-09 |
| LRRC8C | 2,079 | 8,56E-10 |
| FAM198B | 2,075 | 2,62E-09 |
| ANGPT2 | 2,073 | 1,78E-09 |
| CDK14 | 2,068 | 1,38E-07 |
| PI15 | 2,064 | 1,21E-07 |
| SOAT1 | 2,060 | 4,79E-09 |
| LAMB3 | 2,047 | 1,98E-09 |
| CCDC80 | 2,046 | 3,92E-06 |
| IFITM1 | 2,046 | 1,49E-08 |
| KCNS3 | 2,045 | 4,51E-07 |
| IGF2BP2 | 2,043 | 1,91E-07 |
| GNA12 | 2,040 | 3,09E-10 |
| SULF1 | 2,039 | 3,78E-06 |
| FADS2 | 2,036 | 6,69E-08 |
| CDH3 | 2,029 | 1,83E-08 |
| HOXD10 | 2,028 | 3,82E-08 |
| F2R | 2,024 | 6,59E-09 |
| MGP | 2,020 | 2,56E-07 |
| CHN1 | 2,016 | 1,14E-09 |
| GOLM1 | 2,006 | 7,94E-08 |
| SELL | 2,004 | 3,91E-08 |
| ELTD1 | 2,003 | 1,80E-07 |
| LAMC1 | 2,003 | 4,38E-11 |
| GJC1 | 2,001 | 6,67E-06 |
| VCAM1 | 1,997 | 1,11E-07 |
| CLIC4 | 1,984 | 4,17E-09 |
| PSMB9 | 1,983 | 5,05E-09 |
| SYT1 | 1,983 | 4,71E-06 |
| GBP5 | 1,979 | 1,34E-06 |
| EIF2AK2 | 1,977 | 7,10E-09 |
| SRGN | 1,976 | 1,37E-10 |
| FMNL2 | 1,976 | 4,12E-08 |
| MSR1 | 1,971 | 2,21E-07 |
| ITM2C | 1,965 | 1,33E-12 |
| ARPC1B | 1,956 | 8,98E-09 |
| FAT1 | 1,955 | 2,35E-11 |
| SDC2 | 1,955 | 8,29E-10 |
| COL6A3 | 1,949 | 2,04E-07 |
| G0S2 | 1,941 | 1,67E-07 |
| GOLIM4 | 1,940 | 6,70E-09 |
| TMTC1 | 1,936 | 1,12E-06 |
| HLTF | 1,935 | 1,80E-07 |
| RCN1 | 1,934 | 6,67E-05 |
| CCL2 | 1,931 | 7,42E-07 |
| ADAMDEC1 | 1,926 | 1,03E-07 |
| TNFRSF12A | 1,926 | 3,54E-07 |
| DUSP6 | 1,926 | 8,29E-10 |
| GLIPR1 | 1,921 | 2,87E-08 |
| PON3 | 1,920 | 5,17E-06 |
| LAMP3 | 1,914 | 9,21E-06 |
| GBP1 | 1,914 | 5,87E-07 |
| CD14 | 1,910 | 3,54E-11 |
| RGS5 | 1,909 | 1,47E-05 |
| FOXM1 | 1,907 | 9,31E-11 |
| SELE | 1,905 | 1,49E-05 |
| TGFB2 | 1,901 | 4,77E-07 |
| SEMA3A | 1,900 | 9,54E-07 |
| FERMT2 | 1,900 | 9,70E-08 |
| IFI30 | 1,894 | 4,17E-11 |
| SLC2A3 | 1,889 | 2,80E-07 |
| LAPTM4B | 1,886 | 2,68E-10 |
| MYBL2 | 1,886 | 2,33E-11 |
| PTHLH | 1,877 | 1,53E-05 |
| C1QC | 1,876 | 6,46E-09 |
| ECE1 | 1,867 | 5,23E-10 |
| SYT14 | 1,866 | 3,21E-05 |
| TRIP13 | 1,865 | 1,13E-07 |
| PLOD3 | 1,865 | 6,70E-09 |
| RAD51AP1 | 1,863 | 9,83E-09 |
| CEP55 | 1,855 | 7,61E-09 |
| KLHL5 | 1,853 | 8,49E-08 |
| CD200 | 1,853 | 2,71E-05 |
| A2M | 1,852 | 2,25E-07 |
| IFI35 | 1,852 | 7,51E-09 |
| SLC39A14 | 1,852 | 8,98E-09 |
| ENAH | 1,847 | 1,22E-08 |
| SGCE | 1,846 | 4,36E-07 |
| MFAP2 | 1,846 | 4,11E-09 |
| NRP2 | 1,845 | 7,05E-10 |
| KIF14 | 1,845 | 1,89E-07 |
| NELL2 | 1,844 | 1,66E-05 |
| SCHIP1 | 1,844 | 1,61E-09 |
| MINPP1 | 1,843 | 1,45E-07 |
| GUCY1A3 | 1,842 | 6,06E-10 |
| FHL1 | 1,838 | 2,59E-06 |
| CYBB | 1,827 | 1,92E-08 |
| KIF18A | 1,823 | 2,95E-07 |
| KIAA1524 | 1,823 | 1,19E-07 |
| TSPAN15 | 1,818 | 7,88E-07 |
| FAM26E | 1,814 | 1,39E-06 |
| IGSF6 | 1,810 | 2,78E-09 |
| PDPN | 1,793 | 1,04E-08 |
| MCM7 | 1,791 | 1,38E-10 |
| PRSS23 | 1,787 | 5,10E-08 |
| CAV1 | 1,786 | 8,13E-07 |
| PLCB1 | 1,786 | 1,20E-05 |
| LYZ | 1,782 | 6,91E-06 |
| KIAA1324L | 1,781 | 6,42E-07 |
| NUF2 | 1,773 | 3,94E-08 |
| PVRL3 | 1,769 | 3,46E-05 |
| MB21D1 | 1,768 | 1,71E-07 |
| CLMP | 1,765 | 5,62E-05 |
| CDK1 | 1,757 | 1,46E-07 |
| PARP14 | 1,755 | 1,96E-08 |
| CD276 | 1,755 | 5,01E-12 |
| IFIT2 | 1,754 | 7,41E-06 |
| TPM1 | 1,754 | 1,56E-07 |
| DDHD1 | 1,749 | 5,05E-09 |
| PLEK | 1,749 | 3,82E-08 |
| EMP3 | 1,745 | 3,91E-08 |
| CPNE8 | 1,740 | 1,07E-05 |
| KIAA0040 | 1,740 | 7,56E-09 |
| ANTXR1 | 1,737 | 1,29E-08 |
| TPX2 | 1,737 | 4,64E-09 |
| TLR2 | 1,735 | 1,47E-07 |
| CLC | 1,729 | 1,36E-05 |
| MME | 1,723 | 1,42E-05 |
| GPX8 | 1,722 | 1,05E-05 |
| HLA-F | 1,718 | 1,68E-08 |
| FAM46C | 1,717 | 1,75E-07 |
| SCCPDH | 1,714 | 2,71E-05 |
| SNX10 | 1,713 | 1,01E-08 |
| FAR2 | 1,711 | 2,53E-07 |
| ALG1L | 1,711 | 9,81E-07 |
| PTPRM | 1,711 | 7,51E-09 |
| SLC7A7 | 1,709 | 1,40E-11 |
| TOP2A | 1,706 | 1,80E-07 |
| CDC25B | 1,703 | 1,49E-07 |
| ADAM6 | 1,699 | 2,78E-08 |
| PLTP | 1,699 | 2,02E-06 |
| DFNA5 | 1,698 | 2,62E-05 |
| DCN | 1,697 | 3,34E-05 |
| TFRC | 1,695 | 3,58E-08 |
| DPYSL2 | 1,693 | 4,91E-07 |
| TIMP3 | 1,690 | 1,59E-06 |
| HSD17B11 | 1,684 | 3,56E-06 |
| STIL | 1,683 | 8,96E-08 |
| RGS2 | 1,681 | 9,79E-08 |
| PDIA5 | 1,680 | 2,84E-08 |
| KDELR3 | 1,680 | 5,33E-08 |
| BGN | 1,674 | 7,12E-07 |
| NCF2 | 1,673 | 1,01E-07 |
| OLFM1 | 1,670 | 7,22E-05 |
| NEDD9 | 1,670 | 8,31E-08 |
| MYO5A | 1,670 | 2,48E-08 |
| BCL2A1 | 1,668 | 1,84E-08 |
| PPT1 | 1,668 | 2,49E-07 |
| FEZ1 | 1,668 | 5,75E-07 |
| TCN2 | 1,667 | 1,10E-07 |
| TLR4 | 1,666 | 1,12E-10 |
| CENPF | 1,660 | 2,50E-06 |
| GLT8D2 | 1,657 | 1,14E-05 |
| NA | 1,656 | 1,07E-05 |
| TMEM176A | 1,656 | 2,11E-09 |
| LAMA4 | 1,654 | 3,99E-08 |
| CDC20 | 1,653 | 7,31E-10 |
| UHRF1 | 1,653 | 1,92E-08 |
| P4HA1 | 1,653 | 1,35E-05 |
| ETV5 | 1,650 | 1,13E-06 |
| EXO1 | 1,649 | 9,67E-08 |
| NA | 1,639 | 1,98E-05 |
| UBE2T | 1,630 | 1,46E-08 |
| CDCA7L | 1,629 | 8,60E-07 |
| PTPRJ | 1,628 | 6,06E-10 |
| PDCD1LG2 | 1,628 | 1,98E-08 |
| GAL | 1,628 | 8,25E-06 |
| NA | 1,627 | 8,50E-07 |
| MTBP | 1,625 | 7,34E-07 |
| MCTP1 | 1,618 | 3,39E-08 |
| NA | 1,618 | 2,42E-07 |
| SH3PXD2B | 1,617 | 3,51E-08 |
| ZAK | 1,617 | 3,80E-08 |
| IL13RA2 | 1,613 | 3,71E-05 |
| LHFPL2 | 1,613 | 2,66E-11 |
| LRRC8D | 1,612 | 1,31E-08 |
| LPCAT1 | 1,611 | 7,88E-09 |
| RAI14 | 1,607 | 3,19E-07 |
| VIM | 1,607 | 1,50E-07 |
| ENO2 | 1,607 | 5,22E-05 |
| ICAM1 | 1,607 | 9,86E-07 |
| FSCN1 | 1,601 | 4,09E-06 |
| PDE3B | 1,601 | 2,63E-06 |
| HMMR | 1,601 | 4,87E-08 |
| SEMA3D | 1,597 | 2,11E-05 |
| C20orf112 | 1,593 | 2,27E-06 |
| ATAD2 | 1,593 | 2,15E-08 |
| F2RL2 | 1,590 | 2,88E-05 |
| RGS1 | 1,589 | 1,87E-08 |
| NPNT | 1,589 | 1,54E-05 |
| LBH | 1,586 | 3,17E-08 |
| CERS2 | 1,585 | 1,11E-10 |
| ETS1 | 1,583 | 2,47E-07 |
| STEAP2 | 1,581 | 1,73E-05 |
| TMEM47 | 1,581 | 1,17E-05 |
| ARHGAP29 | 1,579 | 6,75E-06 |
| BLM | 1,578 | 4,00E-08 |
| CDH13 | 1,578 | 1,25E-05 |
| CDKN3 | 1,577 | 2,16E-07 |
| PIK3R3 | 1,575 | 2,78E-05 |
| RNF130 | 1,575 | 2,03E-07 |
| FANCI | 1,574 | 1,13E-07 |
| CMBL | 1,573 | 3,77E-05 |
| TAP1 | 1,573 | 3,37E-08 |
| TNFAIP3 | 1,570 | 5,92E-07 |
| ADAM12 | 1,570 | 4,41E-06 |
| LOXL2 | 1,568 | 3,58E-08 |
| DUSP10 | 1,564 | 2,92E-08 |
| ALDH1B1 | 1,563 | 9,86E-08 |
| PTPRK | 1,561 | 1,58E-09 |
| C15orf37 | 1,560 | 3,93E-08 |
| FUT8 | 1,559 | 2,47E-07 |
| DOK3 | 1,559 | 1,66E-07 |
| MIR650 | 1,559 | 6,74E-08 |
| TNFRSF10C | 1,559 | 4,58E-05 |
| CD53 | 1,559 | 7,94E-08 |
| KIF20A | 1,557 | 2,45E-09 |
| C1QB | 1,556 | 4,59E-08 |
| ENPP2 | 1,556 | 1,15E-05 |
| FYN | 1,553 | 1,11E-07 |
| DOCK11 | 1,552 | 1,15E-05 |
| TNFRSF10B | 1,551 | 5,89E-09 |
| PLK1 | 1,550 | 4,15E-09 |
| CDK6 | 1,549 | 8,13E-07 |
| WDR54 | 1,549 | 1,32E-07 |
| RDH10 | 1,547 | 6,30E-05 |
| SOCS3 | 1,546 | 2,28E-09 |
| KIAA0101 | 1,543 | 9,03E-07 |
| C1QA | 1,540 | 3,95E-08 |
| LIFR | 1,539 | 1,52E-05 |
| EDN1 | 1,533 | 5,92E-05 |
| FANCI | 1,532 | 5,36E-07 |
| FCHSD2 | 1,531 | 1,59E-06 |
| AURKA | 1,531 | 2,92E-09 |
| CENPI | 1,530 | 5,44E-07 |
| NXPE3 | 1,530 | 6,97E-06 |
| ADRBK2 | 1,527 | 9,41E-07 |
| PRICKLE1 | 1,526 | 3,93E-07 |
| COLGALT1 | 1,526 | 1,90E-10 |
| LOX | 1,524 | 7,86E-05 |
| ITGA3 | 1,523 | 2,70E-07 |
| ICOS | 1,522 | 2,70E-07 |
| TMEM176B | 1,521 | 3,87E-10 |
| PPFIA1 | 1,521 | 2,91E-06 |
| CENPK | 1,521 | 5,39E-05 |
| ZEB1 | 1,520 | 6,91E-07 |
| IRAK3 | 1,519 | 1,68E-05 |
| RFC3 | 1,519 | 7,35E-08 |
| LAMA3 | 1,519 | 1,47E-09 |
| SLFN11 | 1,518 | 2,95E-05 |
| ANLN | 1,518 | 2,56E-07 |
| F13A1 | 1,517 | 3,87E-05 |
| CTHRC1 | 1,517 | 1,80E-07 |
| PRAME | 1,516 | 4,58E-06 |
| NEK6 | 1,516 | 3,12E-08 |
| CCDC88A | 1,516 | 7,98E-07 |
| FBN1 | 1,515 | 7,95E-06 |
| FYB | 1,515 | 5,59E-06 |
| NCAPH | 1,512 | 8,99E-09 |
| PGM2L1 | 1,508 | 3,59E-06 |
| NAPEPLD | 1,506 | 6,02E-09 |
| TYROBP | 1,506 | 1,78E-10 |
| CD93 | 1,505 | 1,46E-08 |
| SLC39A8 | 1,504 | 2,15E-09 |
| LURAP1L | 1,503 | 7,13E-08 |
| LYN | 1,502 | 3,03E-05 |
| ABCC5 | 1,500 | 3,03E-05 |
| LAMB1 | 1,495 | 3,34E-08 |
| BUB1 | 1,492 | 2,92E-08 |
| CCNB2 | 1,490 | 8,28E-07 |
| CSGALNACT2 | 1,488 | 3,34E-08 |
| FAM69A | 1,487 | 4,03E-06 |
| CALU | 1,487 | 5,12E-08 |
| MCM8 | 1,486 | 3,03E-06 |
| KIF4A | 1,485 | 1,09E-08 |
| NA | 1,484 | 1,54E-06 |
| EPSTI1 | 1,484 | 2,11E-05 |
| MFHAS1 | 1,483 | 5,76E-09 |
| LARP6 | 1,483 | 5,49E-09 |
| MCAM | 1,481 | 3,46E-10 |
| WDHD1 | 1,481 | 3,97E-07 |
| IFNGR1 | 1,481 | 1,64E-07 |
| DLGAP5 | 1,480 | 3,19E-07 |
| MX2 | 1,479 | 5,98E-05 |
| MSN | 1,474 | 2,27E-07 |
| RAB23 | 1,473 | 1,80E-08 |
| MPDZ | 1,473 | 5,74E-07 |
| XPR1 | 1,473 | 8,80E-09 |
| COL4A1 | 1,473 | 3,05E-10 |
| IL24 | 1,472 | 3,03E-06 |
| RARRES3 | 1,471 | 7,97E-05 |
| NNMT | 1,469 | 3,91E-07 |
| MS4A4A | 1,468 | 6,28E-05 |
| OAS2 | 1,468 | 1,19E-05 |
| PRIM2 | 1,468 | 2,07E-06 |
| NA | 1,464 | 4,67E-07 |
| DEPDC1 | 1,461 | 3,11E-06 |
| STAT1 | 1,461 | 1,38E-07 |
| UCP2 | 1,457 | 3,02E-06 |
| ITGA1 | 1,457 | 3,72E-06 |
| PRPS1 | 1,457 | 2,75E-07 |
| PRRX1 | 1,457 | 7,40E-07 |
| ITGB4 | 1,456 | 4,83E-09 |
| TK1 | 1,454 | 4,21E-08 |
| TRIO | 1,453 | 4,57E-08 |
| ATP13A3 | 1,450 | 1,10E-07 |
| DSCC1 | 1,447 | 7,70E-07 |
| BRCA1 | 1,442 | 1,60E-06 |
| MAN1A1 | 1,442 | 5,46E-08 |
| PCDH18 | 1,440 | 2,61E-07 |
| MSH6 | 1,439 | 2,58E-07 |
| KIF2C | 1,437 | 7,97E-09 |
| FKBP14 | 1,435 | 8,73E-06 |
| CDC45 | 1,435 | 4,93E-08 |
| KLF7 | 1,435 | 4,05E-07 |
| BRIP1 | 1,434 | 4,89E-06 |
| ASPM | 1,432 | 1,67E-06 |
| ASF1B | 1,431 | 7,19E-08 |
| VPS13C | 1,430 | 9,48E-07 |
| SLC25A36 | 1,428 | 2,30E-05 |
| STC2 | 1,428 | 8,28E-07 |
| SCYL3 | 1,428 | 1,12E-07 |
| DMXL2 | 1,426 | 9,80E-07 |
| ATP11C | 1,423 | 1,43E-07 |
| KNTC1 | 1,422 | 2,17E-06 |
| SPC25 | 1,422 | 1,28E-05 |
| APP | 1,422 | 9,31E-11 |
| FLNA | 1,422 | 3,34E-08 |
| AKR1B1 | 1,422 | 9,67E-08 |
| PLXNA1 | 1,422 | 2,97E-09 |
| CXCR4 | 1,421 | 7,01E-06 |
| NA | 1,418 | 2,29E-07 |
| ACPL2 | 1,416 | 2,48E-05 |
| TMEM71 | 1,413 | 6,09E-05 |
| TMEM140 | 1,412 | 1,70E-07 |
| FKBP10 | 1,410 | 2,09E-07 |
| STOM | 1,410 | 4,52E-07 |
| ATP1B3 | 1,406 | 1,20E-06 |
| EXT1 | 1,405 | 9,86E-08 |
| ABL2 | 1,404 | 1,57E-08 |
| WIPF1 | 1,403 | 1,92E-08 |
| PDLIM3 | 1,402 | 7,42E-07 |
| TYMS | 1,401 | 1,61E-06 |
| ATP6V1C1 | 1,400 | 5,05E-07 |
| ADCY3 | 1,397 | 4,79E-09 |
| GNG11 | 1,396 | 4,12E-06 |
| ST3GAL5 | 1,396 | 2,95E-07 |
| HELLS | 1,396 | 2,96E-05 |
| COL4A2 | 1,395 | 1,41E-09 |
| CMKLR1 | 1,392 | 3,75E-08 |
| MCM2 | 1,391 | 3,47E-09 |
| E2F7 | 1,390 | 1,93E-08 |
| SPRY4 | 1,389 | 9,47E-08 |
| CHEK1 | 1,386 | 7,98E-07 |
| FBLN5 | 1,384 | 5,32E-07 |
| CDCA5 | 1,382 | 1,91E-08 |
| ENTPD1 | 1,381 | 4,78E-07 |
| PRR11 | 1,380 | 9,54E-07 |
| GUCY1B3 | 1,375 | 2,18E-08 |
| KIRREL | 1,375 | 1,56E-06 |
| SLC1A4 | 1,374 | 3,20E-05 |
| MYADM | 1,371 | 2,18E-06 |
| CXorf36 | 1,371 | 6,31E-07 |
| MTHFD2 | 1,370 | 1,19E-07 |
| GINS2 | 1,369 | 2,24E-06 |
| BUB1B | 1,367 | 1,86E-06 |
| NA | 1,367 | 2,46E-06 |
| MICAL2 | 1,365 | 7,42E-07 |
| TTK | 1,364 | 9,44E-07 |
| FAM60A | 1,364 | 3,68E-07 |
| FZD6 | 1,362 | 1,25E-07 |
| NA | 1,361 | 4,97E-05 |
| DSG2 | 1,360 | 2,06E-07 |
| JADE2 | 1,360 | 5,68E-08 |
| HCP5 | 1,358 | 1,13E-06 |
| TNFSF13B | 1,356 | 1,11E-07 |
| NDC80 | 1,355 | 4,17E-06 |
| RRN3P3 | 1,355 | 1,81E-08 |
| SCRN1 | 1,355 | 5,27E-07 |
| DTX3L | 1,353 | 1,62E-07 |
| MYO10 | 1,352 | 3,03E-07 |
| NCAPD2 | 1,352 | 8,52E-08 |
| EPB41L2 | 1,350 | 2,51E-06 |
| NA | 1,349 | 9,31E-06 |
| PUS7 | 1,349 | 4,26E-05 |
| SLC20A1 | 1,348 | 6,57E-06 |
| CXCR2P1 | 1,348 | 2,03E-05 |
| DENND5A | 1,346 | 2,51E-07 |
| CHKA | 1,345 | 1,70E-07 |
| CENPV | 1,345 | 2,03E-05 |
| DDR2 | 1,344 | 7,67E-07 |
| NUP155 | 1,342 | 6,16E-07 |
| CD79A | 1,341 | 6,25E-06 |
| MNDA | 1,341 | 7,54E-08 |
| CDC42BPA | 1,339 | 2,10E-05 |
| MARVELD1 | 1,339 | 9,80E-07 |
| MYO1B | 1,338 | 9,63E-05 |
| C12orf23 | 1,338 | 1,09E-06 |
| PIGX | 1,336 | 9,36E-06 |
| DCBLD2 | 1,336 | 6,42E-06 |
| TNS4 | 1,336 | 2,51E-06 |
| KIF23 | 1,333 | 2,11E-07 |
| SLC35G1 | 1,331 | 6,32E-05 |
| PRKCA | 1,331 | 1,84E-07 |
| TUSC3 | 1,331 | 1,91E-05 |
| CKAP2L | 1,329 | 9,01E-06 |
| ACVR1 | 1,328 | 2,11E-07 |
| ITPR3 | 1,327 | 4,83E-09 |
| STMN1 | 1,327 | 2,01E-08 |
| FLVCR1 | 1,326 | 1,06E-05 |
| APMAP | 1,325 | 4,20E-06 |
| GLIPR2 | 1,324 | 5,71E-06 |
| VNN1 | 1,324 | 2,37E-07 |
| MMS22L | 1,323 | 2,43E-06 |
| CHST11 | 1,322 | 1,59E-06 |
| HERC5 | 1,322 | 5,61E-06 |
| TMEM2 | 1,322 | 1,14E-08 |
| LAPTM5 | 1,320 | 1,52E-07 |
| SLC25A32 | 1,320 | 2,44E-06 |
| PDGFRB | 1,318 | 6,87E-08 |
| ACSL4 | 1,317 | 4,72E-06 |
| MCM3 | 1,317 | 1,41E-07 |
| CBLB | 1,317 | 1,97E-06 |
| IGFBP4 | 1,314 | 1,96E-08 |
| MTHFD1L | 1,312 | 2,60E-10 |
| PCOLCE | 1,310 | 6,82E-07 |
| PARP9 | 1,309 | 4,35E-06 |
| CD97 | 1,309 | 6,48E-09 |
| SLFN5 | 1,308 | 4,15E-06 |
| AOAH | 1,307 | 1,37E-05 |
| RASSF8 | 1,306 | 1,28E-08 |
| NUAK1 | 1,306 | 1,06E-07 |
| NREP | 1,305 | 4,82E-06 |
| CEP97 | 1,305 | 6,71E-05 |
| SERPINE2 | 1,304 | 8,17E-07 |
| NUDCD1 | 1,304 | 1,15E-06 |
| CTSZ | 1,304 | 6,77E-10 |
| ASAP1 | 1,303 | 1,36E-07 |
| PARVB | 1,302 | 5,76E-11 |
| SASS6 | 1,302 | 2,21E-07 |
| PAG1 | 1,301 | 3,26E-07 |
| TMEM97 | 1,301 | 8,40E-08 |
| IL6 | 1,301 | 7,32E-05 |
| MCM10 | 1,301 | 2,97E-09 |
| CD180 | 1,301 | 3,58E-08 |
| HEXA | 1,299 | 1,10E-09 |
| GBAS | 1,298 | 5,68E-05 |
| HSPA13 | 1,298 | 6,39E-06 |
| SERPINF1 | 1,297 | 2,54E-05 |
| SAV1 | 1,297 | 1,36E-06 |
| APOBEC3F | 1,296 | 2,46E-05 |
| EVI2B | 1,296 | 1,16E-05 |
| FAM111B | 1,296 | 8,38E-05 |
| NDE1 | 1,295 | 1,76E-08 |
| EDNRA | 1,292 | 9,80E-06 |
| MB21D2 | 1,291 | 8,23E-07 |
| CALD1 | 1,291 | 3,18E-06 |
| AURKB | 1,291 | 1,47E-09 |
| CENPE | 1,290 | 3,92E-05 |
| TOPBP1 | 1,289 | 2,58E-06 |
| BICC1 | 1,288 | 1,79E-05 |
| STK17A | 1,288 | 4,12E-06 |
| VRK1 | 1,288 | 4,40E-06 |
| PODXL | 1,288 | 4,83E-09 |
| DST | 1,287 | 5,68E-05 |
| ACTL6A | 1,287 | 9,38E-07 |
| CEP170 | 1,287 | 8,09E-06 |
| MMP11 | 1,286 | 2,83E-05 |
| NRP1 | 1,286 | 1,49E-08 |
| DSN1 | 1,286 | 1,29E-08 |
| PLOD1 | 1,285 | 6,08E-09 |
| LITAF | 1,285 | 1,34E-09 |
| MELK | 1,285 | 9,25E-06 |
| NA | 1,284 | 1,11E-06 |
| MCM6 | 1,284 | 2,74E-06 |
| EMR2 | 1,283 | 2,68E-10 |
| SPATS2 | 1,282 | 5,11E-07 |
| FOXN2 | 1,282 | 3,74E-06 |
| SIX1 | 1,281 | 3,75E-06 |
| MACF1 | 1,281 | 2,28E-07 |
| MFI2 | 1,280 | 6,80E-08 |
| VNN2 | 1,280 | 2,21E-05 |
| NFE2L1 | 1,280 | 6,28E-09 |
| FXR1 | 1,279 | 3,12E-06 |
| TNIP3 | 1,279 | 3,35E-06 |
| GPR34 | 1,278 | 4,59E-06 |
| XRCC2 | 1,276 | 6,23E-06 |
| PILRA | 1,276 | 6,48E-08 |
| CD209 | 1,276 | 3,92E-06 |
| SIRPA | 1,276 | 3,52E-06 |
| PTDSS1 | 1,276 | 1,44E-06 |
| IFI16 | 1,275 | 1,02E-07 |
| CENPO | 1,274 | 2,58E-07 |
| RAD51 | 1,272 | 1,49E-06 |
| PNPT1 | 1,269 | 1,00E-06 |
| CMSS1 | 1,268 | 8,13E-07 |
| PBK | 1,266 | 3,50E-05 |
| TM4SF18 | 1,266 | 8,38E-06 |
| LRP6 | 1,265 | 1,36E-05 |
| SLCO1A2 | 1,265 | 3,19E-05 |
| DHX36 | 1,265 | 6,70E-05 |
| KIF11 | 1,264 | 2,77E-05 |
| RBL1 | 1,264 | 1,51E-06 |
| FGD6 | 1,264 | 3,70E-07 |
| CCNB1 | 1,264 | 1,34E-07 |
| MND1 | 1,263 | 2,81E-06 |
| CERCAM | 1,261 | 2,08E-05 |
| HAS3 | 1,261 | 6,91E-06 |
| SLC29A1 | 1,261 | 9,56E-08 |
| P2RX4 | 1,259 | 3,39E-08 |
| TTYH3 | 1,257 | 2,51E-10 |
| TCF4 | 1,257 | 3,35E-05 |
| PIK3AP1 | 1,257 | 3,44E-09 |
| HEXB | 1,256 | 1,95E-08 |
| PLVAP | 1,255 | 2,36E-07 |
| PRNP | 1,253 | 1,76E-08 |
| ID3 | 1,253 | 2,58E-07 |
| SPAG5 | 1,252 | 5,19E-09 |
| ANXA6 | 1,251 | 2,76E-07 |
| HIST2H2AB | 1,250 | 1,69E-05 |
| RHOJ | 1,249 | 9,56E-08 |
| CEP19 | 1,249 | 5,72E-05 |
| TRIB2 | 1,248 | 2,68E-05 |
| PDE7A | 1,248 | 6,82E-05 |
| ITGAV | 1,248 | 3,77E-05 |
| FLT1 | 1,248 | 1,27E-05 |
| WDR76 | 1,248 | 9,15E-07 |
| SLC35B2 | 1,247 | 2,24E-07 |
| SLC38A6 | 1,247 | 1,37E-05 |
| OIP5 | 1,246 | 4,48E-06 |
| STARD3NL | 1,246 | 1,00E-06 |
| SMC2 | 1,246 | 7,97E-05 |
| FMOD | 1,243 | 7,21E-06 |
| AP2B1 | 1,243 | 6,69E-07 |
| SLC7A6 | 1,243 | 1,65E-06 |
| HIP1 | 1,242 | 3,18E-06 |
| NAV1 | 1,242 | 4,88E-05 |
| LILRB3 | 1,242 | 7,24E-07 |
| ERAP1 | 1,241 | 1,51E-05 |
| ABCC4 | 1,240 | 1,56E-06 |
| GALNT6 | 1,239 | 1,69E-07 |
| CKAP5 | 1,239 | 4,37E-07 |
| HJURP | 1,237 | 1,04E-07 |
| LILRB2 | 1,237 | 1,95E-09 |
| HIST1H2AB | 1,236 | 7,28E-06 |
| NUSAP1 | 1,234 | 1,78E-06 |
| GPRC5B | 1,234 | 1,31E-07 |
| CFB | 1,234 | 7,71E-05 |
| TGFBR2 | 1,233 | 9,67E-08 |
| TINAGL1 | 1,233 | 2,70E-05 |
| FPR2 | 1,233 | 3,52E-06 |
| CHRNA5 | 1,232 | 7,47E-06 |
| MKI67 | 1,231 | 2,59E-06 |
| SLC5A12 | 1,227 | 6,89E-05 |
| NEXN | 1,227 | 6,76E-05 |
| ATAD5 | 1,227 | 2,26E-06 |
| BTN3A2 | 1,224 | 8,02E-06 |
| SSFA2 | 1,223 | 5,87E-08 |
| CASC5 | 1,223 | 1,64E-05 |
| DPY19L4 | 1,221 | 2,42E-06 |
| MICB | 1,221 | 1,06E-07 |
| NA | 1,221 | 9,14E-06 |
| CENPA | 1,220 | 6,60E-08 |
| GRB10 | 1,219 | 1,55E-07 |
| GPR116 | 1,218 | 1,07E-06 |
| NCS1 | 1,217 | 2,80E-07 |
| PANX1 | 1,216 | 8,47E-07 |
| TEAD2 | 1,216 | 7,47E-10 |
| SLC41A2 | 1,214 | 3,34E-06 |
| ELMO1 | 1,214 | 1,16E-06 |
| C11orf24 | 1,214 | 6,93E-09 |
| FBXO5 | 1,213 | 4,06E-07 |
| TSHZ3 | 1,213 | 9,79E-05 |
| EXTL2 | 1,212 | 1,91E-05 |
| PSME4 | 1,210 | 1,08E-06 |
| MRGBP | 1,210 | 2,40E-06 |
| LILRB1 | 1,210 | 3,68E-07 |
| PTP4A3 | 1,210 | 5,46E-09 |
| AHR | 1,210 | 9,43E-06 |
| RFC5 | 1,210 | 1,14E-06 |
| TYMP | 1,209 | 2,97E-07 |
| OLR1 | 1,208 | 2,45E-05 |
| SLAMF8 | 1,208 | 6,94E-09 |
| SNAPC1 | 1,208 | 3,62E-06 |
| SPDL1 | 1,207 | 9,90E-07 |
| NA | 1,206 | 1,60E-06 |
| SLC6A6 | 1,205 | 2,66E-06 |
| SH3BP4 | 1,205 | 5,47E-07 |
| RSRC1 | 1,204 | 8,79E-06 |
| FANCB | 1,204 | 1,43E-06 |
| CPQ | 1,204 | 5,16E-06 |
| BMP2 | 1,203 | 1,58E-05 |
| WARS | 1,202 | 4,03E-05 |
| HPS3 | 1,202 | 5,25E-05 |
| PIM2 | 1,199 | 8,05E-09 |
| NA | 1,199 | 6,77E-08 |
| NUP107 | 1,198 | 1,83E-06 |
| ENG | 1,197 | 8,95E-09 |
| RRM2 | 1,197 | 4,10E-07 |
| NA | 1,196 | 1,33E-06 |
| HLA-DMB | 1,196 | 4,60E-05 |
| SHCBP1 | 1,196 | 5,92E-07 |
| BTN3A2 | 1,195 | 1,45E-06 |
| DCBLD1 | 1,194 | 6,10E-06 |
| TMEM67 | 1,193 | 1,70E-05 |
| NA | 1,193 | 7,61E-07 |
| VOPP1 | 1,193 | 4,11E-05 |
| ZNF827 | 1,192 | 3,21E-07 |
| SYT11 | 1,192 | 6,11E-09 |
| KIF15 | 1,192 | 1,48E-05 |
| TGIF2 | 1,192 | 6,33E-08 |
| PARP1 | 1,188 | 1,17E-07 |
| CKS2 | 1,188 | 1,19E-06 |
| TAGLN | 1,187 | 9,58E-06 |
| KLHL23 | 1,186 | 1,16E-05 |
| LCP2 | 1,185 | 2,27E-07 |
| RUVBL1 | 1,184 | 3,44E-07 |
| CLSPN | 1,184 | 4,86E-07 |
| PTPRC | 1,183 | 1,82E-05 |
| ALMS1 | 1,182 | 1,19E-05 |
| DEK | 1,182 | 9,15E-07 |
| SLC12A2 | 1,182 | 5,25E-06 |
| SGOL2 | 1,181 | 2,05E-06 |
| SLC39A6 | 1,181 | 3,10E-06 |
| VSIG4 | 1,180 | 3,29E-06 |
| AUNIP | 1,178 | 5,41E-08 |
| GZMB | 1,178 | 1,58E-06 |
| RAC2 | 1,175 | 2,05E-08 |
| PSMB8 | 1,175 | 1,36E-06 |
| PRC1 | 1,175 | 1,75E-06 |
| YEATS2 | 1,174 | 1,29E-06 |
| PALM2-AKAP2 | 1,172 | 8,96E-07 |
| SP4 | 1,172 | 1,19E-05 |
| SP110 | 1,172 | 2,35E-07 |
| C1RL | 1,171 | 8,67E-06 |
| ATP8B2 | 1,171 | 2,55E-06 |
| MAD2L2 | 1,171 | 3,13E-09 |
| PTPRG | 1,169 | 1,12E-07 |
| NA | 1,168 | 9,03E-07 |
| VAV2 | 1,166 | 1,11E-05 |
| ANXA5 | 1,165 | 3,21E-07 |
| BFAR | 1,164 | 3,10E-07 |
| RECQL | 1,163 | 8,84E-06 |
| ICAM2 | 1,162 | 3,18E-09 |
| DEPDC1B | 1,162 | 8,90E-08 |
| LIMA1 | 1,161 | 3,27E-07 |
| LILRB4 | 1,161 | 5,71E-10 |
| TWSG1 | 1,160 | 7,75E-06 |
| LCLAT1 | 1,160 | 4,94E-08 |
| C3orf17 | 1,159 | 7,96E-06 |
| SESTD1 | 1,159 | 1,31E-05 |
| CYR61 | 1,159 | 3,42E-05 |
| APBB2 | 1,159 | 1,74E-06 |
| PRIM1 | 1,158 | 3,67E-06 |
| NA | 1,157 | 1,16E-05 |
| APOBEC3G | 1,157 | 5,84E-06 |
| AARS | 1,157 | 7,71E-07 |
| CD163 | 1,155 | 1,43E-06 |
| NA | 1,155 | 3,58E-07 |
| RIF1 | 1,154 | 3,68E-05 |
| BTN3A1 | 1,152 | 1,52E-06 |
| MTERFD1 | 1,152 | 2,42E-06 |
| GRK5 | 1,152 | 7,87E-08 |
| HDGFRP3 | 1,151 | 9,20E-07 |
| SP100 | 1,149 | 3,31E-07 |
| FAM49A | 1,149 | 4,68E-07 |
| PLXNC1 | 1,149 | 2,94E-07 |
| TNFRSF9 | 1,148 | 1,04E-07 |
| ZNF639 | 1,148 | 8,19E-05 |
| NRM | 1,148 | 3,82E-07 |
| THSD1P1 | 1,146 | 1,14E-06 |
| ZC3H12A | 1,145 | 3,39E-07 |
| ITGB1 | 1,145 | 7,61E-09 |
| MCM4 | 1,144 | 5,10E-08 |
| WDR66 | 1,144 | 1,36E-06 |
| GMPS | 1,140 | 4,54E-06 |
| SELPLG | 1,138 | 9,82E-07 |
| GLA | 1,138 | 1,16E-06 |
| PCNA | 1,137 | 1,95E-06 |
| ST8SIA4 | 1,137 | 1,13E-07 |
| PTGFRN | 1,137 | 1,70E-08 |
| IGF2 | 1,136 | 4,77E-06 |
| CKB | 1,136 | 1,63E-05 |
| RTP4 | 1,136 | 6,17E-05 |
| CYP2S1 | 1,135 | 8,59E-05 |
| IKBIP | 1,132 | 2,08E-05 |
| STRIP2 | 1,132 | 4,29E-07 |
| ZEB2 | 1,130 | 1,73E-08 |
| IPO9 | 1,128 | 1,82E-07 |
| NDC1 | 1,125 | 5,32E-06 |
| ADAR | 1,124 | 1,40E-08 |
| MTHFS | 1,123 | 4,77E-07 |
| NRG1 | 1,123 | 1,27E-05 |
| APOLD1 | 1,122 | 3,02E-07 |
| TAPBPL | 1,121 | 1,04E-05 |
| SRPK2 | 1,118 | 6,88E-06 |
| 1-Mar | 1,118 | 8,62E-07 |
| IL32 | 1,117 | 4,15E-05 |
| CD86 | 1,116 | 1,49E-06 |
| ANPEP | 1,115 | 9,63E-08 |
| RAB7L1 | 1,115 | 5,92E-07 |
| PRKDC | 1,115 | 1,16E-05 |
| BRCA2 | 1,115 | 1,51E-05 |
| SLC3A2 | 1,114 | 1,48E-08 |
| CCDC14 | 1,113 | 8,95E-05 |
| CYTH4 | 1,112 | 1,28E-08 |
| SEC62 | 1,111 | 3,54E-06 |
| TRAF5 | 1,111 | 1,88E-06 |
| RHOC | 1,110 | 3,41E-07 |
| CENPQ | 1,109 | 2,14E-05 |
| SP140L | 1,107 | 4,07E-06 |
| UBE2L6 | 1,107 | 1,33E-07 |
| DNMT1 | 1,107 | 4,11E-09 |
| CDK4 | 1,106 | 4,33E-08 |
| MEF2C | 1,105 | 2,93E-06 |
| MTRR | 1,104 | 3,28E-06 |
| LCP1 | 1,104 | 4,34E-05 |
| TXNDC12 | 1,103 | 1,94E-07 |
| CD151 | 1,103 | 6,17E-07 |
| ELOVL5 | 1,102 | 7,23E-07 |
| CLPTM1L | 1,101 | 6,53E-06 |
| LY6E | 1,101 | 2,11E-05 |
| RBMS3 | 1,100 | 2,95E-07 |
| RPS6KA1 | 1,100 | 1,80E-07 |
| STK3 | 1,099 | 3,72E-07 |
| SLC41A1 | 1,098 | 2,67E-07 |
| FANCL | 1,097 | 2,83E-05 |
| CAV2 | 1,096 | 2,25E-06 |
| PRKD1 | 1,096 | 4,41E-05 |
| OLFML1 | 1,095 | 4,53E-06 |
| SLC5A3 | 1,095 | 4,83E-06 |
| DYNC1I1 | 1,095 | 2,92E-06 |
| POLA2 | 1,095 | 2,44E-07 |
| ANP32E | 1,094 | 6,09E-07 |
| ARSB | 1,094 | 7,12E-09 |
| NA | 1,093 | 3,04E-05 |
| C11orf82 | 1,092 | 6,24E-05 |
| RFTN2 | 1,092 | 4,89E-06 |
| CNPY3 | 1,089 | 4,67E-07 |
| 3-Sep | 1,089 | 2,61E-05 |
| ZNFX1 | 1,088 | 1,44E-06 |
| MTR | 1,087 | 3,36E-06 |
| GSG2 | 1,086 | 1,92E-08 |
| CDCA8 | 1,086 | 3,95E-06 |
| CDK2 | 1,085 | 3,17E-07 |
| PRKD3 | 1,084 | 1,39E-05 |
| CCDC77 | 1,084 | 1,31E-05 |
| RNF168 | 1,083 | 8,37E-05 |
| MASTL | 1,082 | 2,06E-07 |
| ATP2C1 | 1,082 | 9,88E-06 |
| CHML | 1,081 | 6,59E-06 |
| CD27 | 1,080 | 5,85E-05 |
| MCM5 | 1,080 | 9,25E-08 |
| ZNF146 | 1,078 | 8,71E-05 |
| CMTM7 | 1,078 | 8,67E-06 |
| XRN2 | 1,077 | 8,82E-06 |
| STX2 | 1,077 | 3,36E-06 |
| AEBP1 | 1,073 | 3,18E-07 |
| KBTBD2 | 1,073 | 2,24E-06 |
| IRF1 | 1,072 | 4,14E-06 |
| PON2 | 1,072 | 1,49E-07 |
| CLUAP1 | 1,072 | 9,11E-05 |
| ITGB3 | 1,070 | 1,55E-08 |
| GCA | 1,070 | 4,78E-05 |
| PTK7 | 1,070 | 1,34E-09 |
| ENPEP | 1,070 | 5,59E-05 |
| CASD1 | 1,069 | 3,59E-05 |
| YWHAH | 1,066 | 1,86E-08 |
| CDC42SE2 | 1,066 | 9,47E-07 |
| IFIH1 | 1,066 | 4,12E-05 |
| RPL15 | 1,066 | 3,88E-06 |
| USP18 | 1,065 | 1,07E-05 |
| GNS | 1,065 | 1,24E-07 |
| RAB34 | 1,063 | 4,64E-09 |
| GMNN | 1,061 | 2,59E-07 |
| INSR | 1,061 | 9,79E-06 |
| DNAJC9 | 1,060 | 2,04E-06 |
| PIGK | 1,059 | 5,99E-06 |
| COL5A2 | 1,059 | 3,24E-07 |
| NOP2 | 1,058 | 4,28E-06 |
| CRISPLD2 | 1,058 | 1,33E-07 |
| IRF8 | 1,058 | 5,96E-06 |
| HIST1H2AL | 1,057 | 5,28E-06 |
| NID2 | 1,057 | 1,09E-06 |
| SLC12A7 | 1,056 | 4,74E-06 |
| TMEM39A | 1,056 | 2,23E-06 |
| IFT22 | 1,056 | 1,30E-07 |
| CTSC | 1,056 | 1,93E-06 |
| NA | 1,056 | 4,55E-08 |
| HAUS6 | 1,056 | 1,05E-05 |
| CCNA2 | 1,055 | 5,87E-07 |
| RAB8B | 1,055 | 9,03E-06 |
| FGF2 | 1,054 | 2,24E-05 |
| CSGALNACT1 | 1,052 | 4,60E-07 |
| CYSTM1 | 1,052 | 2,35E-06 |
| SLC9B2 | 1,051 | 6,92E-05 |
| NCAPG | 1,050 | 6,09E-06 |
| NEDD1 | 1,050 | 1,72E-05 |
| NFIL3 | 1,050 | 9,46E-05 |
| ADAM17 | 1,049 | 6,65E-06 |
| NT5DC3 | 1,049 | 7,19E-05 |
| POC5 | 1,049 | 2,87E-05 |
| FAM89A | 1,048 | 2,63E-06 |
| NUP62CL | 1,047 | 2,28E-05 |
| DNAJC10 | 1,047 | 4,14E-05 |
| ORAI2 | 1,047 | 9,60E-05 |
| SMURF2 | 1,046 | 4,12E-06 |
| PCNX | 1,046 | 3,25E-06 |
| GIMAP4 | 1,045 | 2,85E-06 |
| GMFG | 1,045 | 1,61E-07 |
| CCP110 | 1,045 | 1,99E-05 |
| RAD54B | 1,044 | 3,63E-07 |
| GORAB | 1,044 | 5,32E-07 |
| TMEM209 | 1,041 | 1,86E-05 |
| NCAPG2 | 1,041 | 7,24E-05 |
| TBL1XR1 | 1,041 | 7,53E-06 |
| NSL1 | 1,040 | 4,91E-06 |
| CDCA2 | 1,039 | 3,71E-06 |
| NASP | 1,038 | 2,49E-06 |
| ADAMTS1 | 1,035 | 5,29E-05 |
| PSMB4 | 1,034 | 1,63E-07 |
| DNAJB11 | 1,033 | 7,35E-06 |
| C5AR1 | 1,032 | 8,18E-07 |
| RHPN2 | 1,032 | 2,65E-05 |
| CCT5 | 1,032 | 6,64E-06 |
| SPRED1 | 1,032 | 2,19E-05 |
| PRR5L | 1,031 | 1,30E-05 |
| TMEM138 | 1,031 | 1,08E-06 |
| ARL13B | 1,031 | 9,15E-05 |
| TIMELESS | 1,030 | 2,42E-07 |
| NINJ1 | 1,030 | 9,85E-05 |
| FOLR2 | 1,030 | 1,63E-05 |
| PTK2 | 1,029 | 8,58E-07 |
| KRTCAP3 | 1,029 | 2,17E-05 |
| B2M | 1,029 | 9,15E-07 |
| PAPD7 | 1,027 | 7,00E-06 |
| OLFML3 | 1,027 | 1,77E-05 |
| DARS2 | 1,026 | 2,34E-06 |
| NA | 1,025 | 3,82E-05 |
| SELM | 1,025 | 1,70E-08 |
| ITGA4 | 1,024 | 5,01E-06 |
| BCAM | 1,023 | 4,45E-05 |
| SLC43A2 | 1,021 | 1,49E-08 |
| ABCC9 | 1,020 | 3,21E-05 |
| SDK1 | 1,019 | 8,58E-08 |
| LRP8 | 1,019 | 1,51E-05 |
| BIRC5 | 1,019 | 5,83E-07 |
| EFNB1 | 1,019 | 3,64E-07 |
| SECTM1 | 1,018 | 8,59E-05 |
| SOD2 | 1,017 | 1,44E-05 |
| TMEFF1 | 1,017 | 9,95E-08 |
| FADS3 | 1,017 | 5,21E-06 |
| FAM92A1P2 | 1,016 | 1,23E-06 |
| FGL2 | 1,016 | 8,99E-06 |
| GLI3 | 1,014 | 5,96E-05 |
| SNAI2 | 1,013 | 3,54E-06 |
| CTSS | 1,012 | 3,93E-05 |
| HIST1H3H | 1,011 | 6,13E-07 |
| JOSD1 | 1,010 | 1,76E-08 |
| RCBTB1 | 1,009 | 1,85E-05 |
| DIEXF | 1,009 | 1,31E-06 |
| SCD5 | 1,008 | 4,94E-06 |
| NA | 1,008 | 6,95E-05 |
| C3orf52 | 1,008 | 3,50E-07 |
| MAD2L1 | 1,007 | 2,17E-05 |
| SOCS1 | 1,006 | 2,03E-07 |
| HEATR1 | 1,006 | 5,23E-06 |
| SKA1 | 1,006 | 8,40E-06 |
| CKAP2 | 1,005 | 3,03E-05 |
| KLHL42 | 1,005 | 5,49E-07 |
| CENPH | 1,005 | 7,19E-05 |
| C16orf80 | 1,005 | 2,95E-07 |
| HEATR1 | 1,003 | 2,10E-05 |
| RUNX2 | 1,003 | 3,36E-06 |
| TLR1 | 1,003 | 1,87E-05 |
| NA | 1,003 | 9,34E-06 |
| LAIR1 | 1,002 | 5,15E-07 |
| CDCA3 | 1,001 | 6,01E-06 |
| GPN3 | 1,000 | 5,24E-05 |
|  |  |  |

| **Gene Symbol** | **Expression Variation - ESCC / Adj Mucosa (logFC)** | **Adjusted p value** |
| --- | --- | --- |
| CRISP3 | -4,616 | 4,11E-07 |
| TMPRSS11B | -4,434 | 1,03E-06 |
| DYNAP | -4,097 | 3,03E-05 |
| ACER1 | -3,596 | 2,51E-10 |
| ENDOU | -3,441 | 3,94E-08 |
| CLCA4 | -3,408 | 7,54E-05 |
| PPP1R3C | -3,282 | 7,21E-06 |
| KRT78 | -3,248 | 7,19E-06 |
| GYS2 | -3,236 | 4,11E-09 |
| PLA2G2A | -3,197 | 2,95E-07 |
| SLURP1 | -3,112 | 8,75E-07 |
| HPGD | -3,013 | 1,16E-06 |
| SPINK5 | -2,904 | 5,38E-05 |
| EPGN | -2,902 | 3,00E-05 |
| PADI1 | -2,901 | 3,99E-08 |
| SCEL | -2,886 | 1,89E-05 |
| SH3BGRL2 | -2,874 | 1,25E-07 |
| GCOM1 | -2,770 | 2,58E-07 |
| CWH43 | -2,724 | 3,06E-05 |
| MT1G | -2,712 | 6,76E-06 |
| DPCR1 | -2,552 | 1,02E-10 |
| RAET1E | -2,524 | 1,28E-07 |
| ANXA9 | -2,523 | 5,07E-06 |
| ECM1 | -2,510 | 1,67E-06 |
| FAM3B | -2,488 | 4,09E-05 |
| AIF1L | -2,482 | 1,99E-08 |
| SCIN | -2,456 | 3,59E-06 |
| SLC16A6 | -2,433 | 4,95E-09 |
| FAM3D | -2,419 | 1,48E-07 |
| GDPD3 | -2,406 | 7,85E-07 |
| NCCRP1 | -2,368 | 4,33E-05 |
| MUC15 | -2,346 | 5,54E-05 |
| GGTA1P | -2,277 | 9,04E-10 |
| BNIPL | -2,268 | 3,92E-06 |
| LYPD2 | -2,245 | 5,94E-08 |
| CRABP2 | -2,240 | 8,92E-05 |
| IL18 | -2,234 | 6,39E-08 |
| SASH1 | -2,216 | 1,11E-07 |
| TPRG1 | -2,214 | 4,44E-07 |
| PSCA | -2,206 | 8,64E-06 |
| SCNN1B | -2,198 | 8,25E-08 |
| CYP4B1 | -2,191 | 3,36E-06 |
| PRSS27 | -2,187 | 9,79E-05 |
| GBP6 | -2,186 | 3,24E-06 |
| TMPRSS2 | -2,161 | 9,47E-05 |
| CAB39L | -2,161 | 5,41E-08 |
| RDH12 | -2,144 | 1,55E-05 |
| KLK12 | -2,136 | 6,37E-06 |
| BARX2 | -2,126 | 6,78E-08 |
| FUT3 | -2,111 | 3,83E-06 |
| ABLIM3 | -2,106 | 1,16E-07 |
| MGLL | -2,098 | 1,98E-08 |
| PAX9 | -2,075 | 4,57E-08 |
| CAPN5 | -2,072 | 9,13E-08 |
| CGNL1 | -2,064 | 8,01E-09 |
| CLIC3 | -2,058 | 1,80E-07 |
| ATP13A4 | -2,036 | 3,34E-05 |
| CPA4 | -2,031 | 7,29E-06 |
| BLNK | -2,015 | 2,74E-06 |
| UBL3 | -1,985 | 9,96E-10 |
| PPL | -1,980 | 2,17E-07 |
| DLG2 | -1,980 | 2,14E-08 |
| BBOX1 | -1,959 | 5,31E-05 |
| RPSAP58 | -1,952 | 7,46E-05 |
| KAT2B | -1,951 | 1,03E-07 |
| C1orf177 | -1,940 | 4,78E-08 |
| FUT6 | -1,934 | 3,58E-08 |
| SYNPO2L | -1,923 | 9,70E-06 |
| MAB21L3 | -1,922 | 1,44E-05 |
| CD207 | -1,920 | 7,03E-08 |
| TCP11L2 | -1,915 | 1,10E-06 |
| CA13 | -1,904 | 7,94E-08 |
| CTTNBP2 | -1,901 | 6,06E-10 |
| TFAP2B | -1,896 | 8,74E-11 |
| ALOX15B | -1,883 | 7,20E-07 |
| VSIG2 | -1,866 | 2,07E-07 |
| CHAC1 | -1,861 | 4,32E-05 |
| ZNF431 | -1,846 | 6,24E-07 |
| C15orf48 | -1,845 | 2,51E-06 |
| ZNF185 | -1,822 | 1,98E-06 |
| FNDC4 | -1,815 | 6,68E-10 |
| CYP2J2 | -1,813 | 2,36E-07 |
| ALOX12 | -1,807 | 7,88E-09 |
| CYP2E1 | -1,806 | 9,03E-05 |
| CYP4F22 | -1,802 | 1,37E-05 |
| C2orf54 | -1,789 | 1,67E-07 |
| KLF8 | -1,774 | 2,67E-06 |
| LMO7 | -1,774 | 1,82E-06 |
| LNX1 | -1,768 | 1,01E-07 |
| LPIN1 | -1,762 | 1,40E-06 |
| EPB41L4A | -1,761 | 1,51E-06 |
| FAM46B | -1,758 | 2,27E-07 |
| DUSP5 | -1,756 | 3,38E-06 |
| ZNF426 | -1,742 | 1,03E-05 |
| C1orf116 | -1,742 | 1,62E-07 |
| RRAGD | -1,740 | 1,51E-05 |
| ACPP | -1,731 | 1,14E-05 |
| SULT2B1 | -1,723 | 8,70E-05 |
| PTK6 | -1,716 | 8,83E-08 |
| ARHGAP10 | -1,716 | 1,95E-08 |
| RBP7 | -1,709 | 8,98E-07 |
| ST3GAL4 | -1,705 | 2,97E-09 |
| EHD3 | -1,705 | 2,14E-06 |
| GATM | -1,699 | 3,39E-06 |
| NUCB2 | -1,684 | 7,31E-10 |
| C4orf3 | -1,683 | 1,45E-08 |
| ATP13A4 | -1,679 | 4,18E-06 |
| CAMK2N1 | -1,674 | 5,82E-06 |
| EVPL | -1,670 | 4,84E-09 |
| NDUFA4L2 | -1,669 | 2,62E-05 |
| EPS8L1 | -1,667 | 6,28E-08 |
| SORT1 | -1,665 | 3,00E-05 |
| NIPAL1 | -1,653 | 2,67E-07 |
| TP53I3 | -1,643 | 1,77E-07 |
| PHYHD1 | -1,636 | 6,16E-08 |
| EXPH5 | -1,634 | 2,65E-08 |
| MYO5B | -1,633 | 6,76E-06 |
| SLC39A2 | -1,631 | 1,33E-05 |
| MPZL3 | -1,630 | 2,11E-05 |
| PRSS3 | -1,608 | 1,98E-05 |
| NA | -1,607 | 1,65E-07 |
| GPD1L | -1,605 | 2,03E-07 |
| MXD1 | -1,592 | 2,84E-05 |
| OTOP3 | -1,592 | 4,03E-06 |
| TJP3 | -1,592 | 4,69E-06 |
| TRIP10 | -1,589 | 2,00E-07 |
| S100A14 | -1,579 | 7,15E-06 |
| ATG9B | -1,572 | 1,45E-07 |
| GCNT3 | -1,569 | 5,63E-05 |
| MC5R | -1,568 | 7,00E-06 |
| TTC22 | -1,568 | 9,28E-08 |
| IKZF2 | -1,564 | 3,73E-06 |
| PIK3C2G | -1,555 | 8,77E-06 |
| DOPEY2 | -1,550 | 1,49E-07 |
| C18orf25 | -1,545 | 1,70E-08 |
| SHROOM3 | -1,543 | 4,94E-08 |
| GALNT12 | -1,539 | 9,71E-06 |
| CITED2 | -1,532 | 1,23E-06 |
| SLC16A9 | -1,532 | 2,77E-05 |
| SLC6A4 | -1,525 | 2,37E-06 |
| GGT6 | -1,498 | 2,02E-06 |
| DDAH1 | -1,497 | 2,24E-06 |
| PHACTR2 | -1,497 | 3,19E-07 |
| ABLIM1 | -1,495 | 2,10E-07 |
| MACC1 | -1,494 | 5,27E-06 |
| WNK4 | -1,490 | 3,27E-09 |
| SLC24A3 | -1,488 | 3,03E-07 |
| DHRS1 | -1,487 | 4,48E-05 |
| TMEM40 | -1,483 | 1,06E-05 |
| CYP4F12 | -1,482 | 3,13E-07 |
| C4orf3 | -1,481 | 1,20E-05 |
| LAMB4 | -1,480 | 1,70E-07 |
| VAT1 | -1,473 | 6,93E-09 |
| CH25H | -1,471 | 6,01E-05 |
| EPS8L2 | -1,450 | 4,43E-09 |
| GRHL1 | -1,446 | 7,00E-06 |
| MPP7 | -1,442 | 4,13E-05 |
| PAQR5 | -1,441 | 5,86E-06 |
| GATSL3 | -1,441 | 4,85E-08 |
| GULP1 | -1,438 | 5,29E-05 |
| CES2 | -1,437 | 8,43E-08 |
| TP53INP2 | -1,434 | 8,61E-06 |
| SMAGP | -1,432 | 3,55E-06 |
| CXCR2 | -1,422 | 5,37E-06 |
| GRHL3 | -1,420 | 9,41E-07 |
| NMRK1 | -1,419 | 1,74E-05 |
| RAB11FIP1 | -1,415 | 1,38E-05 |
| ANKRD37 | -1,413 | 5,37E-05 |
| IL34 | -1,413 | 2,03E-07 |
| TEC | -1,412 | 7,95E-09 |
| NMU | -1,412 | 1,33E-06 |
| UPK3B | -1,409 | 4,52E-09 |
| FAM129B | -1,408 | 5,17E-08 |
| EMP1 | -1,406 | 2,39E-06 |
| ZNF750 | -1,404 | 1,11E-05 |
| SIM2 | -1,398 | 1,67E-07 |
| GPT2 | -1,397 | 2,39E-06 |
| WNK1 | -1,391 | 1,03E-07 |
| RASGEF1B | -1,385 | 5,46E-06 |
| CD1E | -1,378 | 1,82E-05 |
| GCHFR | -1,374 | 5,13E-07 |
| SNX24 | -1,373 | 1,06E-07 |
| TTC39A | -1,373 | 2,69E-05 |
| DEPTOR | -1,368 | 2,15E-06 |
| PLEKHA7 | -1,366 | 2,61E-07 |
| NA | -1,361 | 9,16E-11 |
| SFTA2 | -1,359 | 1,98E-09 |
| FAM63A | -1,357 | 1,62E-07 |
| CYP11A1 | -1,344 | 2,01E-06 |
| GAB2 | -1,338 | 8,41E-10 |
| THSD4 | -1,330 | 1,25E-05 |
| GRPEL2 | -1,323 | 1,16E-05 |
| HLF | -1,322 | 1,91E-05 |
| CPEB3 | -1,317 | 2,07E-09 |
| BMS1P20 | -1,313 | 2,95E-06 |
| ALDH9A1 | -1,313 | 1,10E-07 |
| TIAM1 | -1,312 | 3,99E-06 |
| UNC93A | -1,306 | 9,92E-08 |
| MAPT | -1,301 | 1,78E-10 |
| SLC13A4 | -1,300 | 1,81E-08 |
| EPHX2 | -1,299 | 2,11E-07 |
| ANKRD35 | -1,298 | 1,65E-08 |
| ETFDH | -1,296 | 5,05E-07 |
| USP6NL | -1,296 | 4,20E-07 |
| PMM1 | -1,295 | 3,98E-08 |
| SMIM5 | -1,294 | 6,70E-09 |
| 5-Sep | -1,293 | 4,82E-06 |
| RORA | -1,292 | 9,64E-07 |
| SDCBP2 | -1,291 | 6,54E-05 |
| TOM1L2 | -1,290 | 1,76E-08 |
| VSIG10 | -1,285 | 1,51E-06 |
| PDLIM2 | -1,270 | 2,22E-07 |
| BTBD11 | -1,270 | 1,09E-06 |
| PIM1 | -1,267 | 1,09E-06 |
| MLLT4 | -1,265 | 2,24E-06 |
| TMEM154 | -1,264 | 1,17E-05 |
| CD1A | -1,256 | 1,02E-05 |
| HOPX | -1,256 | 2,25E-07 |
| SLC35C1 | -1,255 | 1,36E-05 |
| RANBP9 | -1,255 | 1,48E-07 |
| ABHD5 | -1,255 | 5,95E-07 |
| STK39 | -1,252 | 2,31E-07 |
| ARHGAP32 | -1,250 | 2,42E-07 |
| MTMR10 | -1,249 | 1,04E-08 |
| TMEM79 | -1,246 | 1,03E-05 |
| GMDS | -1,242 | 2,15E-06 |
| CSTA | -1,238 | 2,49E-05 |
| MALL | -1,236 | 4,07E-06 |
| C3orf67 | -1,231 | 9,83E-09 |
| MOSPD1 | -1,224 | 1,22E-06 |
| PITX1 | -1,223 | 6,65E-06 |
| AGFG2 | -1,220 | 2,84E-08 |
| MAML3 | -1,220 | 8,40E-08 |
| EPHA1 | -1,218 | 6,28E-08 |
| ECHDC2 | -1,208 | 2,86E-06 |
| OBFC1 | -1,207 | 4,06E-09 |
| BOC | -1,200 | 1,62E-07 |
| CCNG2 | -1,199 | 1,45E-06 |
| GLTP | -1,192 | 9,85E-06 |
| UACA | -1,191 | 6,79E-08 |
| ZNF681 | -1,190 | 3,89E-05 |
| PDCD4 | -1,189 | 2,88E-05 |
| DEGS2 | -1,185 | 1,44E-08 |
| ARHGAP27 | -1,185 | 3,17E-08 |
| CYP7B1 | -1,184 | 1,18E-05 |
| SECISBP2L | -1,183 | 3,16E-07 |
| AIM1L | -1,182 | 5,50E-06 |
| BSPRY | -1,180 | 9,67E-07 |
| GPR126 | -1,175 | 5,35E-05 |
| NPAS2 | -1,174 | 3,46E-06 |
| BLVRB | -1,174 | 3,02E-05 |
| TMEM80 | -1,172 | 9,28E-08 |
| CPEB4 | -1,166 | 1,37E-05 |
| AHNAK | -1,162 | 2,48E-08 |
| PDZD2 | -1,161 | 7,33E-05 |
| ITPR2 | -1,157 | 9,01E-06 |
| ACOX1 | -1,153 | 3,51E-06 |
| TOM1L2 | -1,148 | 1,45E-07 |
| ATP6V1D | -1,140 | 1,66E-05 |
| CYP2C19 | -1,138 | 6,10E-06 |
| KANK1 | -1,136 | 2,48E-07 |
| ZNF365 | -1,135 | 4,27E-06 |
| NA | -1,128 | 1,22E-05 |
| DAPP1 | -1,125 | 4,82E-06 |
| SNX16 | -1,120 | 1,43E-06 |
| RMND5B | -1,119 | 4,61E-07 |
| TMOD3 | -1,116 | 6,96E-07 |
| DOCK9 | -1,116 | 5,75E-07 |
| MYO6 | -1,114 | 8,01E-06 |
| ZNF737 | -1,111 | 2,47E-07 |
| TM7SF2 | -1,110 | 5,54E-06 |
| MAPK3 | -1,109 | 1,46E-07 |
| PLXNA2 | -1,108 | 4,29E-06 |
| TPCN1 | -1,104 | 6,93E-09 |
| WDR26 | -1,104 | 2,74E-08 |
| RIOK3 | -1,103 | 4,57E-08 |
| CTNNBIP1 | -1,100 | 7,41E-07 |
| ULK3 | -1,093 | 2,95E-07 |
| PHLDA1 | -1,091 | 1,32E-05 |
| VASN | -1,089 | 1,40E-06 |
| DENND2C | -1,087 | 1,95E-07 |
| PLEKHM1 | -1,087 | 1,51E-06 |
| PAQR8 | -1,085 | 1,42E-10 |
| TNFAIP8L3 | -1,082 | 1,65E-06 |
| ACOX3 | -1,082 | 2,06E-07 |
| RORC | -1,081 | 2,92E-08 |
| NR1D1 | -1,080 | 6,02E-05 |
| CNN3 | -1,080 | 7,88E-07 |
| VPS4B | -1,079 | 1,67E-07 |
| IL12A | -1,072 | 1,76E-07 |
| EPHA6 | -1,067 | 8,92E-05 |
| TOM1 | -1,066 | 3,18E-06 |
| RAB5B | -1,066 | 9,04E-08 |
| ZFP36 | -1,066 | 2,74E-06 |
| SLC6A1 | -1,064 | 5,13E-07 |
| RNF141 | -1,059 | 2,27E-07 |
| LGALS3 | -1,056 | 1,26E-05 |
| CNKSR3 | -1,054 | 6,93E-06 |
| KIAA1468 | -1,054 | 2,53E-07 |
| KRTAP20-1 | -1,053 | 1,25E-05 |
| PCDH1 | -1,051 | 1,95E-05 |
| NLRX1 | -1,050 | 2,41E-08 |
| ARHGAP5-AS1 | -1,049 | 3,61E-07 |
| CLVS1 | -1,048 | 2,07E-07 |
| ZBED2 | -1,047 | 7,47E-05 |
| FAM221A | -1,047 | 2,05E-07 |
| PPDPF | -1,043 | 1,10E-07 |
| TP53AIP1 | -1,040 | 8,24E-05 |
| GAB1 | -1,040 | 3,74E-05 |
| RASAL2 | -1,032 | 8,98E-07 |
| UNC13B | -1,030 | 6,65E-05 |
| IL22RA1 | -1,030 | 7,42E-07 |
| KRT32 | -1,029 | 8,17E-05 |
| ZDHHC13 | -1,029 | 1,03E-07 |
| WWC1 | -1,029 | 5,63E-06 |
| PELI1 | -1,028 | 5,22E-05 |
| SNX9 | -1,028 | 7,83E-06 |
| SLC7A4 | -1,026 | 9,85E-06 |
| PLEKHA6 | -1,026 | 1,61E-06 |
| ZC4H2 | -1,023 | 1,41E-06 |
| USP46 | -1,020 | 1,93E-07 |
| LRP10 | -1,018 | 2,44E-06 |
| XYLT1 | -1,017 | 4,86E-06 |
| CDH17 | -1,016 | 6,51E-05 |
| MBNL3 | -1,016 | 1,36E-05 |
| ESPL1 | -1,015 | 4,24E-07 |
| VLDLR | -1,015 | 7,88E-07 |
| TSPAN6 | -1,014 | 1,30E-05 |
| TAB3 | -1,012 | 1,53E-06 |
| VPS37B | -1,011 | 2,79E-07 |
| MAGI3 | -1,010 | 6,92E-07 |
| RNF208 | -1,008 | 9,92E-08 |
| TYRO3 | -1,005 | 1,49E-07 |
| SERAC1 | -1,004 | 1,19E-05 |
| DUOXA1 | -1,004 | 1,90E-05 |
| PLAGL1 | -1,002 | 3,71E-06 |
| ITPKC | -1,001 | 3,68E-06 |
