# Supplementary material for: Esophageal squamous cell carcinoma transcriptome reveals the effect of FOXM1 on patient outcome through novel PIK3R3 mediated activation of PI3K signaling pathway
Source: Oncotarget. 2018 Mar 30;9(24):16634–47. doi: 10.18632/oncotarget.24621 (PMC5908275; doi:10.18632/oncotarget.24621)
Supplement: Supplementary file 3 [file oncotarget-09-16634-s003.doc]

Supplementary Table 2: Major regulator gene outcome of transcriptional network analysis.

| **Transcriptional Factor (Gene Symbol)** | **Adjusted p value** |
| --- | --- |
| ZNF281 | 3,60E-108 |
| PAX9 | 2,30E-100 |
| TEAD2 | 2,70E-085 |
| HOPX | 2,90E-083 |
| E2F7 | 1,80E-082 |
| RUNX1 | 6,60E-067 |
| WHSC1 | 8,10E-067 |
| BARX2 | 6,00E-064 |
| LITAF | 1,80E-061 |
| MYBL2 | 2,20E-061 |
| PRRX1 | 3,90E-059 |
| FOXM1 | 3,30E-057 |
| TFAP2B | 4,80E-052 |
| FOXF2 | 1,50E-048 |
| ZEB1 | 5,70E-043 |
| ZNF296 | 6,30E-042 |
| GRHL3 | 9,50E-042 |
| TFAP4 | 2,50E-039 |
| KLF11 | 8,30E-039 |
| KLF7 | 8,30E-038 |
| ZNF426 | 4,90E-037 |
| GRHL1 | 1,60E-033 |
| MXD1 | 1,30E-032 |
| ETS1 | 2,90E-030 |
| TSHZ3 | 4,00E-029 |
| STAT1 | 6,50E-029 |
| TFEC | 6,50E-029 |
| ZBTB10 | 6,50E-029 |
| TBX6 | 8,30E-029 |
| CEBPB | 6,20E-028 |
| SP4 | 1,40E-027 |
| PITX1 | 1,90E-027 |
| FOXI2 | 2,90E-027 |
| ZNF124 | 5,30E-027 |
| NFE2L1 | 8,50E-027 |
| ZNF431 | 8,50E-027 |
| EGR2 | 1,00E-026 |
| GLIS2 | 1,30E-026 |
| ZNF554 | 6,40E-026 |
| SNAI1 | 2,20E-025 |
| ZNF639 | 2,90E-025 |
| RELB | 8,60E-025 |
| ZNF827 | 9,60E-024 |
| ETV5 | 2,80E-023 |
| CERS2 | 6,40E-023 |
| KLF8 | 2,20E-022 |
| IKZF2 | 2,30E-022 |
| NFATC1 | 3,40E-022 |
| RORA | 1,20E-020 |
| CEBPD | 2,60E-020 |
| ZNF737 | 2,00E-019 |
| TCFL5 | 2,80E-019 |
| NPAS2 | 7,10E-019 |
| FOXI1 | 8,20E-019 |
| TEAD4 | 1,60E-018 |
| ZNF425 | 5,10E-018 |
| HEY1 | 9,50E-018 |
| NR2F2 | 2,30E-017 |
| TGIF2 | 1,40E-016 |
| ZBTB7A | 1,40E-016 |
| TOX | 2,50E-016 |
| ZNF532 | 3,40E-016 |
| FOXN2 | 5,90E-016 |
| E2F3 | 2,20E-014 |
| SPZ1 | 2,30E-014 |
| TSC22D4 | 7,20E-014 |
| RORC | 1,00E-013 |
| CREB5 | 9,40E-013 |
| ZEB2 | 1,40E-012 |
| OSR2 | 4,70E-012 |
| MEIS1 | 7,80E-012 |
| ETS2 | 3,80E-011 |
| HOXD10 | 3,80E-011 |
| SP100 | 4,10E-011 |
| TCF7L1 | 4,10E-011 |
| DDIT3 | 4,20E-011 |
| SP110 | 4,30E-011 |
| GLI3 | 6,50E-011 |
| ZNF274 | 1,20E-010 |
| TCF4 | 1,30E-010 |
| PRDM1 | 1,70E-010 |
| NFIX | 4,80E-010 |
| ZNF681 | 6,90E-010 |
| ERG | 7,80E-010 |
| MAFF | 7,80E-010 |
| THAP5 | 7,90E-010 |
| ERF | 1,00E-009 |
| NR5A2 | 1,00E-009 |
| IRF3 | 1,60E-009 |
| ARID5A | 3,80E-009 |
| TBX2 | 1,80E-008 |
| SNAI2 | 2,20E-008 |
| TCF3 | 3,20E-008 |
| FOXO4 | 5,70E-008 |
| NFE2L3 | 6,40E-008 |
| IRF1 | 7,30E-008 |
| TBX3 | 7,30E-008 |
| NFIL3 | 9,30E-008 |
| EBF1 | 1,20E-007 |
| TERF1 | 1,50E-007 |
| NFIA | 2,10E-007 |
| SP140L | 2,20E-007 |
| ID4 | 2,60E-007 |
| ZBED2 | 3,00E-007 |
| RUNX3 | 4,30E-007 |
| AFF4 | 8,50E-007 |
| ID3 | 9,90E-007 |
| E2F1 | 1,10E-006 |
| NR1D1 | 1,10E-006 |
| PLAGL1 | 1,10E-006 |
| TFDP2 | 2,30E-006 |
| SIX1 | 2,80E-006 |
| ETV4 | 3,40E-006 |
| ETV6 | 4,40E-006 |
| ZNF132 | 6,00E-006 |
| ATF2 | 7,10E-006 |
| ZNF135 | 8,60E-006 |
| MSC | 1,00E-005 |
| HLF | 1,20E-005 |
| RERE | 1,20E-005 |
| NFKB2 | 1,70E-005 |
| HOXB7 | 1,80E-005 |
| ZNF101 | 2,50E-005 |
| RARA | 2,80E-005 |
| EHF | 3,00E-005 |
| ZNF22 | 3,40E-005 |
| AR | 3,70E-005 |
| HOXC13 | 3,70E-005 |
| KLF10 | 4,10E-005 |
| SOX4 | 5,50E-005 |
| HMGA2 | 6,90E-005 |
| DNAJC2 | 9,20E-005 |
| DMRT2 | 1,20E-004 |
| IRF4 | 1,20E-004 |
| AHR | 1,50E-004 |
| TSC22D2 | 1,80E-004 |
| PKNOX1 | 2,50E-004 |
| TFAM | 2,80E-004 |
| RREB1 | 3,00E-004 |
| BHLHE41 | 3,50E-004 |
| OVOL1 | 3,50E-004 |
| ZKSCAN5 | 4,10E-004 |
| ZNF148 | 4,10E-004 |
| ZNF367 | 5,00E-004 |
| ZNF3 | 7,20E-004 |
| ZHX3 | 7,30E-004 |
| RARG | 7,60E-004 |
| FEZF2 | 1,40E-003 |
| NR1I3 | 1,40E-003 |
| PATZ1 | 1,40E-003 |
| ZNF684 | 1,40E-003 |
| DLX6 | 1,80E-003 |
| NFYB | 1,80E-003 |
| HMGB2 | 2,30E-003 |
| MXI1 | 3,40E-003 |
| ZNF682 | 3,40E-003 |
| ZNF563 | 4,60E-003 |
| ZNF770 | 5,00E-003 |
| CBFB | 5,60E-003 |
| ARNT2 | 8,50E-003 |
| ZNF131 | 9,50E-003 |
| EAF2 | 1,30E-002 |
| ZNF286A | 1,30E-002 |
| MBD2 | 1,60E-002 |
| BBX | 1,80E-002 |
| HOXC9 | 2,00E-002 |
| ZNF200 | 2,90E-002 |
| CREM | 3,00E-002 |
| ELK4 | 3,20E-002 |
| HOXA2 | 3,20E-002 |
| ZNF254 | 3,20E-002 |
| ZSCAN25 | 3,20E-002 |
| ZNF669 | 4,20E-002 |
| EVX1 | 4,50E-002 |
| ZFPM2 | 4,80E-002 |
| ZNF44 | 4,80E-002 |
