# Supplementary material for: Esophageal squamous cell carcinoma transcriptome reveals the effect of FOXM1 on patient outcome through novel PIK3R3 mediated activation of PI3K signaling pathway
Source: Oncotarget. 2018 Mar 30;9(24):16634–47. doi: 10.18632/oncotarget.24621 (PMC5908275; doi:10.18632/oncotarget.24621)
Supplement: Supplementary file 4 [file oncotarget-09-16634-s004.doc]

Supplementary Table 4: Differentially expressed genes (DEG) after *FOXM1* silencing in ESCC-derived TE-1 cell line.

| **Gene Symbol** | **Fold Change (linear)** |
| --- | --- |
| GOLGA6L5 | 3.98 |
| MT1IP | 3.52 |
| KIR2DS4 | 3.39 |
| OR6C3 | 3 |
| KRTAP21-1 | 2.59 |
| OR1N1 | 2.57 |
| KRT6B | 2.4 |
| ZNF678 | 2.27 |
| KIR3DL1 | 2.26 |
| BRD7P3 | 2.25 |
| RNASE3 | 2.25 |
| IFITM3 | 2.24 |
| ACSM2B | 2.22 |
| ZNF267 | 2.17 |
| MLLT6 | 2.16 |
| KRTAP4-12 | 2.12 |
| DYNLL1 | 2.12 |
| CAPNS2 | 2.11 |
| CYP2A7 | 2.07 |
| HTR1A | 2.01 |
| KRTAP6-2 | 1.99 |
| NDUFAF2 | 1.98 |
| ST13P4 | 1.98 |
| MT1M | 1.97 |
| NDUFB1P1 | 1.96 |
| FAM35A | 1.95 |
| ZNF616 | 1.93 |
| ZNF525 | 1.91 |
| EMC6 | 1.91 |
| IFNK | 1.9 |
| FAM216B | 1.89 |
| OR4M2 | 1.88 |
| PPP1R27 | 1.87 |
| ZNF468 | 1.87 |
| OR8S1 | 1.85 |
| NOLC1 | 1.85 |
| FIBIN | 1.84 |
| CYP4A22 | 1.84 |
| POM121L10P | 1.84 |
| DPH3P1 | 1.82 |
| MT1JP | 1.82 |
| FLJ38773 | 1.81 |
| OR4D10 | 1.8 |
| ADAM21 | 1.77 |
| CCDC144A | 1.77 |
| HIST1H2AJ | 1.76 |
| OR10A4 | 1.76 |
| OR4D11 | 1.75 |
| CCR10 | 1.74 |
| ZDHHC16 | 1.74 |
| KIRREL3-AS3 | 1.74 |
| MAGEL2 | 1.74 |
| LINC00282 | 1.74 |
| CCL8 | 1.73 |
| ATP5H | 1.73 |
| ZRSR2 | 1.72 |
| STX12 | 1.71 |
| OR4K1 | 1.68 |
| ZW10 | 1.67 |
| MRGPRX1 | 1.67 |
| GOLGA8B | 1.67 |
| ZNF83 | 1.66 |
| GK3P | 1.65 |
| PRH1-PRR4 | 1.65 |
| SPDYE1 | 1.65 |
| TRAPPC2 | 1.64 |
| WNT8B | 1.64 |
| OR1L8 | 1.63 |
| TMEM215 | 1.63 |
| HIST1H2BE | 1.62 |
| PSG8 | 1.62 |
| KRTAP21-2 | 1.62 |
| C9orf41 | 1.61 |
| ZNF273 | 1.61 |
| ZNF419 | 1.61 |
| CALB2 | 1.6 |
| OR7C2 | 1.6 |
| GANAB | 1.59 |
| TPM3P9 | 1.59 |
| RPS19 | 1.59 |
| RHOXF2 | 1.59 |
| BICD2 | 1.58 |
| C9orf163 | 1.58 |
| HSD17B7P2 | 1.58 |
| CYP1B1 | 1.58 |
| ZSCAN29 | 1.58 |
| OR10H4 | 1.57 |
| OR8B8 | 1.57 |
| GPR22 | 1.57 |
| OR13J1 | 1.57 |
| ZNF701 | 1.56 |
| TOM1L2 | 1.56 |
| DEFA6 | 1.56 |
| GSTA2 | 1.56 |
| RGS19 | 1.55 |
| HABP4 | 1.55 |
| OR5M3 | 1.55 |
| RSPH10B | 1.55 |
| PLDN | 1.55 |
| NXF2B | 1.55 |
| CATSPER2 | 1.55 |
| LOC147727 | 1.55 |
| ZNF271 | 1.54 |
| MICAL2 | 1.54 |
| KRTAP13-3 | 1.53 |
| MC3R | 1.53 |
| MTM1 | 1.53 |
| PSD3 | 1.53 |
| ZBTB8OS | 1.53 |
| ADRA1D | 1.53 |
| TPI1 | 1.53 |
| BRWD1-IT2 | 1.53 |
| LOC100129831 | 1.52 |
| PRR23C | 1.52 |
| KPNA5 | 1.52 |
| ANP32D | 1.52 |
| LOC286359 | 1.52 |
| MIP | 1.52 |
| OR6C76 | 1.52 |
| FLRT3 | 1.51 |
| HSDL2 | 1.51 |
| ARHGAP21 | 1.51 |
| FFAR2 | 1.51 |
| ERVK-7 | 1.51 |
| PRDM9 | 1.51 |
| MPZL3 | 1.51 |
| DEGS2 | 1.51 |

| **Gene Symbol** | **Fold Change (linear)** |
| --- | --- |
| OR4N2 | -4.77 |
| ANKRD36B | -4.09 |
| FOXM1 | -3.95 |
| LCE2D | -3.83 |
| MMP23B | -3.3 |
| TCEAL3 | -2.78 |
| PRY | -2.73 |
| TUBA1B | -2.72 |
| C14orf142 | -2.47 |
| TUBA3E | -2.46 |
| RPL15 | -2.28 |
| CPN1 | -2.27 |
| OR10H1 | -2.26 |
| KRTAP20-1 | -2.24 |
| RPS10P7 | -2.23 |
| PLAC8 | -2.23 |
| FCER1A | -2.22 |
| GABBR1 | -2.21 |
| RHD | -2.13 |
| NFYB | -2.1 |
| PRSS1 | -2.07 |
| TFPI2 | -1.99 |
| CCNG1 | -1.99 |
| CDKN3 | -1.98 |
| STX11 | -1.96 |
| SLC26A2 | -1.96 |
| POT1 | -1.92 |
| UAP1 | -1.92 |
| PIK3R3 | -1.91 |
| ANP32E | -1.91 |
| MRS2 | -1.86 |
| RHEB | -1.81 |
| PRSS35 | -1.81 |
| TAS2R1 | -1.77 |
| YBX1 | -1.77 |
| RRAGC | -1.76 |
| KRTAP9-9 | -1.76 |
| EIF2S2 | -1.75 |
| AZGP1 | -1.74 |
| GFPT1 | -1.73 |
| LSM14B | -1.72 |
| TMSB4X | -1.72 |
| VPS37C | -1.72 |
| ANXA2P1 | -1.71 |
| SLC41A1 | -1.7 |
| CARD16 | -1.7 |
| GUSBP1 | -1.7 |
| CEP19 | -1.69 |
| XCL1 | -1.68 |
| KCNA10 | -1.68 |
| WASF1 | -1.67 |
| C9orf96 | -1.66 |
| MED6 | -1.65 |
| TAS2R4 | -1.65 |
| ASCL3 | -1.65 |
| SELL | -1.65 |
| SPOPL | -1.63 |
| IL10RB | -1.63 |
| AASDH | -1.63 |
| OR6C1 | -1.63 |
| C17orf78 | -1.63 |
| ERI1 | -1.62 |
| FIGN | -1.61 |
| ST3GAL5 | -1.6 |
| IFNA17 | -1.6 |
| TDGF1P3 | -1.59 |
| OR5M9 | -1.59 |
| GRAMD3 | -1.59 |
| C12orf23 | -1.58 |
| QKI | -1.58 |
| PVRIG | -1.58 |
| DMRTC1B | -1.57 |
| GNG11 | -1.57 |
| KRT86 | -1.57 |
| OR52N1 | -1.57 |
| PTGER3 | -1.57 |
| RBMY1J | -1.57 |
| LCE3C | -1.57 |
| SLC35B4 | -1.54 |
| PPIC | -1.54 |
| REEP1 | -1.53 |
| RBM22 | -1.53 |
| OR56A1 | -1.53 |
| F13A1 | -1.53 |
| C11orf44 | -1.52 |
| RRAGD | -1.52 |
| RNF144B | -1.52 |
| PROKR2 | -1.52 |
| OR10AD1 | -1.52 |
| SLC25A4 | -1.52 |
| TMPRSS11E | -1.52 |
| MREG | -1.52 |
| UCHL5 | -1.52 |
| AKR1C1 | -1.51 |
| UBE2E3 | -1.51 |
